# BBS4 regulates the expression and secretion of FSTL1, a protein that participates in ciliogenesis and the differentiation of 3T3-L1

**DOI:** 10.1038/s41598-017-10330-0

**Published:** 2017-08-29

**Authors:** Victoria Prieto-Echagüe, Sukanya Lodh, Laura Colman, Natalia Bobba, Leonardo Santos, Nicholas Katsanis, Carlos Escande, Norann A. Zaghloul, Jose L. Badano

**Affiliations:** 1 Human Molecular Genetics Laboratory, Institut Pasteur de Montevideo, Mataojo 2020, Montevideo, CP11400 Uruguay; 2 INDICyO Institutional Program, Institut Pasteur de Montevideo, Mataojo 2020, Montevideo, CP11400 Uruguay; 3 Division of Endocrinology, Diabetes, and Nutrition, University of Maryland School of Medicine, Baltimore, MD 21201 USA; 4 Metabolic Diseases and Aging Laboratory, Institut Pasteur de Montevideo, Mataojo 2020, Montevideo, CP11400 Uruguay; 5 Department of Cell Biology and Center for Human Disease Modeling, Duke University Medical Center, Durham, NC 27710 USA

## Abstract

Bardet-Biedl syndrome is a model ciliopathy. Although the characterization of BBS proteins has evidenced their involvement in cilia, extraciliary functions for some of these proteins are also being recognized. Importantly, understanding both cilia and cilia-independent functions of the BBS proteins is key to fully dissect the cellular basis of the syndrome. Here we characterize a functional interaction between BBS4 and the secreted protein FSTL1, a protein linked to adipogenesis and inflammation among other functions. We show that BBS4 and cilia regulate FSTL1 mRNA levels, but BBS4 also modulates FSTL1 secretion. Moreover, we show that FSTL1 is a novel regulator of ciliogenesis thus underscoring a regulatory loop between FSTL1 and cilia. Finally, our data indicate that BBS4, cilia and FSTL1 are coordinated during the differentiation of 3T3-L1 cells and that FSTL1 plays a role in this process, at least in part, by modulating ciliogenesis. Therefore, our findings are relevant to fully understand the development of BBS-associated phenotypes such as obesity.

## Introduction

The ciliopathies are a group of human genetic diseases characterized by an overlapping set of phenotypes including cystic kidney disease, retinal degeneration, central nervous defects, polydactyly, diabetes and obesity. This group of disorders presents a common cellular defect: problems in the formation, maintenance and/or function of primary cilia^1–3^. These cellular organelles have been shown to concentrate receptors for a number of paracrine signaling pathways and to participate in sensing and transducing mechanical and chemical cues^4, 5^.

One pleiotropic ciliopathy is Bardet-Biedl syndrome (BBS), where patients present, with variable penetrance, the majority of phenotypes that have been associated with cilia dysfunction^6^. To date, 21 BBS genes have been identified and for the subset of which there has been a functional characterization, the corresponding proteins were associated with the formation and function of cilia^7–13^. Most BBS proteins localize to the base of cilia, the basal body, and can also enter the cilium. A complex of BBS proteins, termed the BBSome, composed of BBS1, 2, 4, 5, 7, 8, 9, and 18/BBIP1/BBIP10 plays a role in vesicle trafficking, transporting ciliary components to the base of the cilium and its interior^14–17^. Other BBS proteins participate in the assembly (BBS6, 10, 12)^15, 18, 19^ and the recruitment (BBS3) of the BBSome to the ciliary membrane^16^, or regulate entrance into the cilium (BBS17)^13^. The BBS proteins have been shown to participate in the regulation of cilia/basal body-associated signaling pathways such as Wnt and Shh^20–22^. In addition, multiple reports support a broader role for the BBS proteins in intracellular trafficking. For example, knockdown of different Bbs genes in zebrafish results in defective melanosome transport and BBS proteins transport the insulin and leptin receptors to the plasma membrane^23–25^. We have shown recently that BBS1 and BBS4 regulate endosomal trafficking of the Notch receptor and its recycling to the plasma membrane^26^. Therefore, understanding the role of BBS proteins and the BBSome, both in the cilium and outside of it, is critical to dissect the cellular basis of BBS.

One hallmark of BBS is obesity, which is thought to have two major components. A hypothalamic/neuro-endocrine dysfunction is thought to be critical in the development of obesity in the ciliopathies as feeding/satiety signaling is altered, likely due to the mislocalization of signaling receptors on neuronal cilia. Recent data is also highlighting an important role of the BBS proteins and cilia in maintaining peripheral tissue homeostasis, particularly in adipose tissue^10, 27–29^. Several BBS proteins have been shown to change their abundance during adipogenesis while cilia are lost in mature adipocytes^30, 31^. Depletion of BBS10 and BBS12 results in impaired ciliogenesis in differentiating adipocytes and increased adipogenesis^31^ while BBS4 was also shown to directly affect adipocyte proliferation and differentiation^32^. However, the mechanisms by which BBS proteins influence adipocyte differentiation remain to be elucidated.

Here we investigated a functional interaction between BBS4 and follistatin-like 1 (FSTL1). *FSTL1* was identified originally as a TGF-β1 regulated gene in a mouse osteoblastic cell line and encodes for a secreted glycoprotein^33^, downregulation of which correlates with myocyte and adipocyte differentiation^34, 35^. In addition, FSTL1 has also been proposed to be a regulator of inflammation and may play a role in inflammation related to obesity and insulin resistance^36–38^. Therefore, FSTL1 has been linked to processes potentially relevant to the pathogenesis of the BBS phenotype, particularly obesity. Here we show that both BBS4 and, more broadly, cilia, regulate the levels of secreted FSTL1 but through discrete mechanisms. While cilia dysfunction results in a reduction in *FSTL1* mRNA levels, knockdown of BBS4 affects both *FSTL1* mRNA and the secretion of the protein. We show that disrupting BBS4 function results in accumulation of FSTL1 in lysosomes, where it is degraded. Importantly, we also report that FSTL1 is not only regulated by the cilium but in turn can modulate ciliogenesis in a cell non-autonomous manner. Finally, our data indicate that BBS4, FSTL1 and the cilium are co-regulated during the differentiation of 3T3-L1 pre-adipocytes, and this process can be affected by modulating the levels of FSTL1. Taken together, our data further support a role for the BBS proteins in intracellular trafficking. Moreover, we report a novel function for FSTL1 in the regulation of ciliogenesis and demonstrate a role for FSTL1 in modulating the differentiation of 3T3-L1 cells.

## Results

### BBS4 knockdown results in a reduction of FSTL1 intracellular and secreted levels

BBS4 contains a series of tetratricopeptide repeats (TPRs) arranged in tandem, a motif typically involved in mediating protein-protein interactions^39, 40^, and is incorporated into the BBSome at the end of the assembly process^18^. Thus, it could play a role acting as an adaptor between the BBSome and its targets^10^. Through a yeast two-hybrid screen using full length BBS4 as bait we previously identified PCM1 and RPN10, thus providing insight into the function of BBS4 and the BBSome complex^20, 40^. In that original screen we also isolated a prey construct containing 762 bp of the *FSTL1* coding region cloned in frame with the myristoylation domain of the pMyr vector and encoding 253 out of the 308 residues of FSTL1 (from residue E56 to the stop codon; reference sequence NM_007085, NP_009016; Fig. S1).

**Figure 1:**
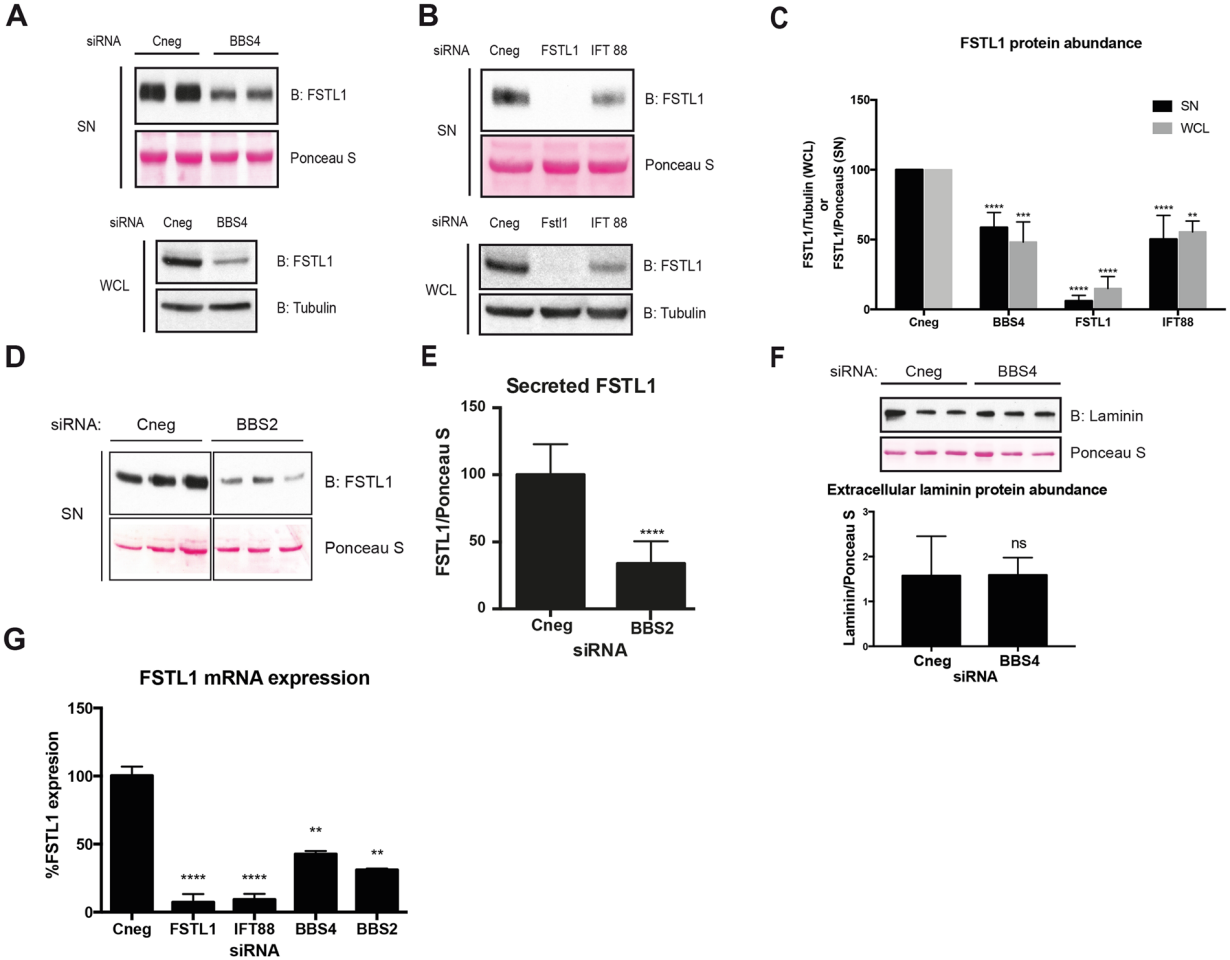
Cilia, BBS4 and BBS2 knockdown result in a reduction of intracellular and secreted FSTL1. **(A)** hTERT-RPE1 cells were transfected with control siRNA (Cneg) or siRNA *BBS4* to produce BBS4 knock-down (BBS4). Secreted FSTL1 and intracellular FSTL1 levels were analyzed in cell culture media (SN) and whole cell lysates (WCL) respectively by Western blot using anti-FSTL1. Full-length blots are shown in Figure S7A. **(B)** hTERT-RPE1 cells were transfected with siRNA to target *FSTL1* and *IFT88* or siRNA Cneg. Secreted FSTL1 and intracellular FSTL1 were analyzed in SN and WCL respectively by Western blot using anti-FSTL1. In both **A** and **B** Ponceau S staining of an unspecific blotted protein and anti-α tubulin were used as loading controls to normalize FSTL1 abundance. Full-length gels are shown in Figure S7D. **(C)** Quantitative representation of densitometry readings of 3 combined experiments shown in (A) and (B). BBS4-KD cells show reduced levels of both intra- and extracellular FSTL1. **(D)** hTERT-RPE1 cells were transfected with siRNA BBS2 or siRNA Cneg and secreted FSTL1 was analyzed by Western blot anti-FSTL1 and Ponceau S staining was used to normalize. The Cneg and BBS2 KD set of lanes were cropped from the same blot. Full-length blots are shown in Figure S7E. **(E)** Densitometry readings of Western blot shown in (D). The results shown are representative of two independent experiments. **(F)** SN of hTERT-RPE1 cells transfected with siRNA *BBS4* were analyzed by Western blot with anti-Laminin and Ponceau S staining to normalize (upper panel). Densitometry readings of the western blot showed that BBS4-KD does not affect Laminin secretion (lower panel). The full-length blot is shown in Figure S7F. **(G)** qRT-PCR was performed to analyze FSTL1 gene expression in hTERT-RPE1 cells transfected with the following siRNAs: Cneg, *BBS4*, *FSTL1*, *IFT88* and *BBS2*. FSTL1 mRNA levels are represented as % expression relative to control siRNA (Cneg)-transfected cells. *FSTL1* expression is reduced by BBS2 and BBS4 KD cells as well as by IFT88/cilia knockdown. Data from at least two independent experiments, designed with biological duplicates, and three technical replicates were normalized relative to Cneg siRNA transfected cells and combined for the analysis. In all cases, error bars represent standard deviation. **: *P* = 0,001–0,01; ***: *P* = 0,0001–0,001 and ****: *P* < 0,0001, ANOVA or t-test.

As mentioned above, FSTL1 has been shown to participate in processes likely relevant to the pathogenesis of BBS. Thus, here we focused on determining whether there is a functional link between BBS4 and FSTL1. We first assessed FSTL1 mRNA and protein levels in cells with depleted BBS4. We used a double stranded RNA oligonucleotide (dsRNA) to specifically target *BBS4* by siRNA in hTERT-RPE1 cells (Fig. S2A,B). We chose this ciliated cell line because i) a secretome analysis showed that hTERT-RPE1 cells express and secrete FSTL1^41^ and ii) these cells have been used to study ciliogenesis^17^. We evaluated the levels of FSTL1 48 hours after BBS4 knockdown (KD) observing a significant decrease in FSTL1 abundance, both intracellular and secreted (Fig. 1A and C). To test whether this effect was linked to the role of the BBS proteins in primary cilia, we targeted the intraflagellar protein IFT88 using our previously validated dsRNA^42^ (Fig. S2A). *IFT88* KD resulted in an approximately 50% reduction in both intracellular and secreted FSTL1 (Fig. 1B,C). To study whether this function of BBS4 occurs in the context of the BBSome, we targeted the complex core protein BBS2^17, 18^. Similarly, FSTL1 secretion was reduced by 60% in BBS2 KD cells (Fig. 1D,E). In contrast, the secretion of laminin, an unrelated extracellular matrix constituent, was not altered by BBS4 KD (Fig. 1F). Thus, our results suggest a specific effect of BBS4, BBS2 and cilia on FSTL1 levels.

The decrease in FSTL1 intracellular protein levels could be explained by changes in the levels of transcript. Using real time quantitative PCR (RTqPCR) we found a 60% reduction of *FSTL1* mRNA in *BBS4* KD cells and a 70% and 90% reduction upon *BBS2* and *IFT88* KD respectively (Fig. 1G). Taken together, our results indicate that BBS2, BBS4 and IFT88/cilia regulate the levels of *FSTL1* mRNA and thus affect the amount of protein in the extracellular space.

### BBS4 and cilia regulate FSTL1 levels by different mechanisms

Since we observed that cilia are important for *FSTL1* gene expression, depleting the BBS proteins could impact *FSTL1* expression by affecting ciliary function. However, while knockdown of BBS4 and IFT88 produced a comparable reduction in FSTL1 protein abundance (50 to 60%; Fig. 1C), the IFT88 knockdown produced a 90% reduction on *FSTL1* mRNA levels compared to the 50% reduction caused by knocking down BBS4 (Fig. 1G). Thus, BBS4 could regulate FSTL1 levels through additional mechanisms.

**Figure 2:**
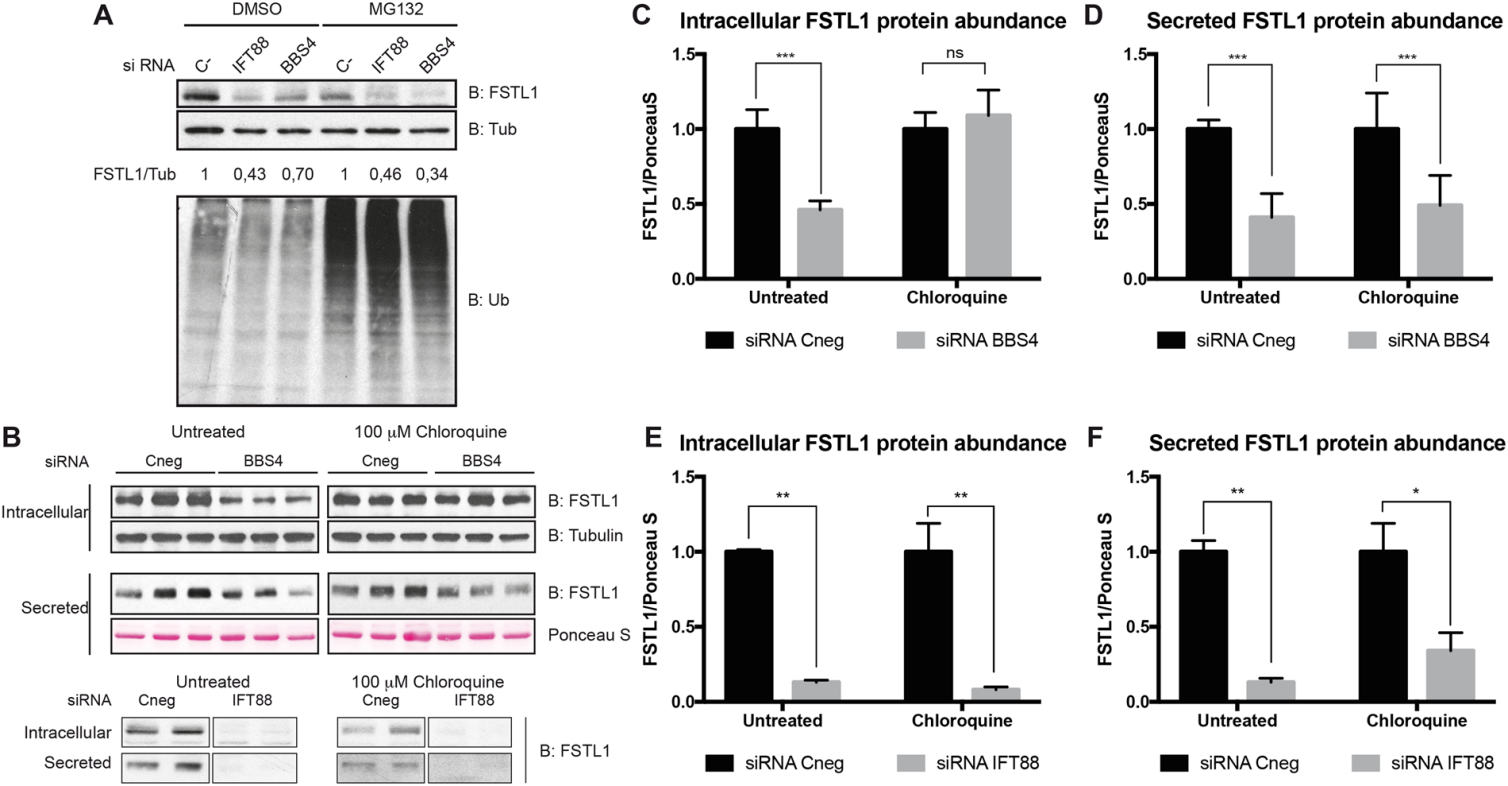
Lysosome inhibition rescues the FSTL1 degradation induced by BBS4 knockdown. **(A)** hTERT-RPE1 cells were transfected with siRNA Cneg, siRNA *IFT88* or siRNA *BBS4* for 48 hours and incubated for 6 hours with MG132. Cell lysates were analyzed by Western blot anti-FSTL1, anti α tubulin and anti-Ubiquitin. Full-length blots are shown in Figure S8A. **(B)** hTERT-RPE1 cells were transfected with siRNA Cneg, siRNA *BBS4* (top panel) or siRNA *IFT88* (bottom panel; for each condition, lanes cropped from the same gel are shown) during 24 hours and incubated with chloroquine for an additional 24 hours. Full-length blots are shown in Figure S8B. **(C-F)** Quantification of experiment shown in (**B**). Densitometry readings were used to calculate ratios of FSTL1/Tubulin to quantitate intracellular FSTL1 and FSTL1/Ponceau S to measure secreted FSTL1. Chloroquine treatment rescued intracellular levels of FSTL1 without affecting its extracellular abundance. Results shown are representative of three independent experiments performed with biological triplicates or duplicates as shown. Error bars represent standard deviation and ns: P > 0.05; *: *P* = 0,01–0,05; **: *P* = 0,001–0,01; ***: *P* = 0,0001–0,001, ANOVA test.

BBS4 has been shown to participate in protein turnover^20, 43^ and trafficking^26, 44^. Thus, we tested whether FSTL1 protein stability was altered in BBS4 KD cells. We therefore treated BBS4 and IFT88 KD cells with the proteasome inhibitor MG132. Inhibiting proteasome-mediated degradation (Fig. 2A lower panel) did not produce a significant increase in FSTL1 levels (Fig. 2A upper panel). Next, we established a working dose of chloroquine to inhibit lysosomal degradation of proteins as evidenced by the accumulation of LC3BII (Fig. S3). Chloroquine treatment restored intracellular FSTL1 levels in BBS4 KD cells to that of controls but did not affect the levels of secreted FSTL1 (Fig. 2B upper panel, 2 C and 2D). In contrast, chloroquine did not rescue the IFT88 KD-mediated drop in FSTL1 intracellular levels (Fig. 2B lower panel, 2E and 2 F). Taken together our data suggest that the decrease in FSTL1 secretion caused by BBS4 depletion has two main components: i) a likely cilia-mediated effect of BBS4 regulating *FSTL1* mRNA levels and ii) a role whereby BBS4 depletion promotes the degradation of FSTL1 in the lysosome.

**Figure 3:**
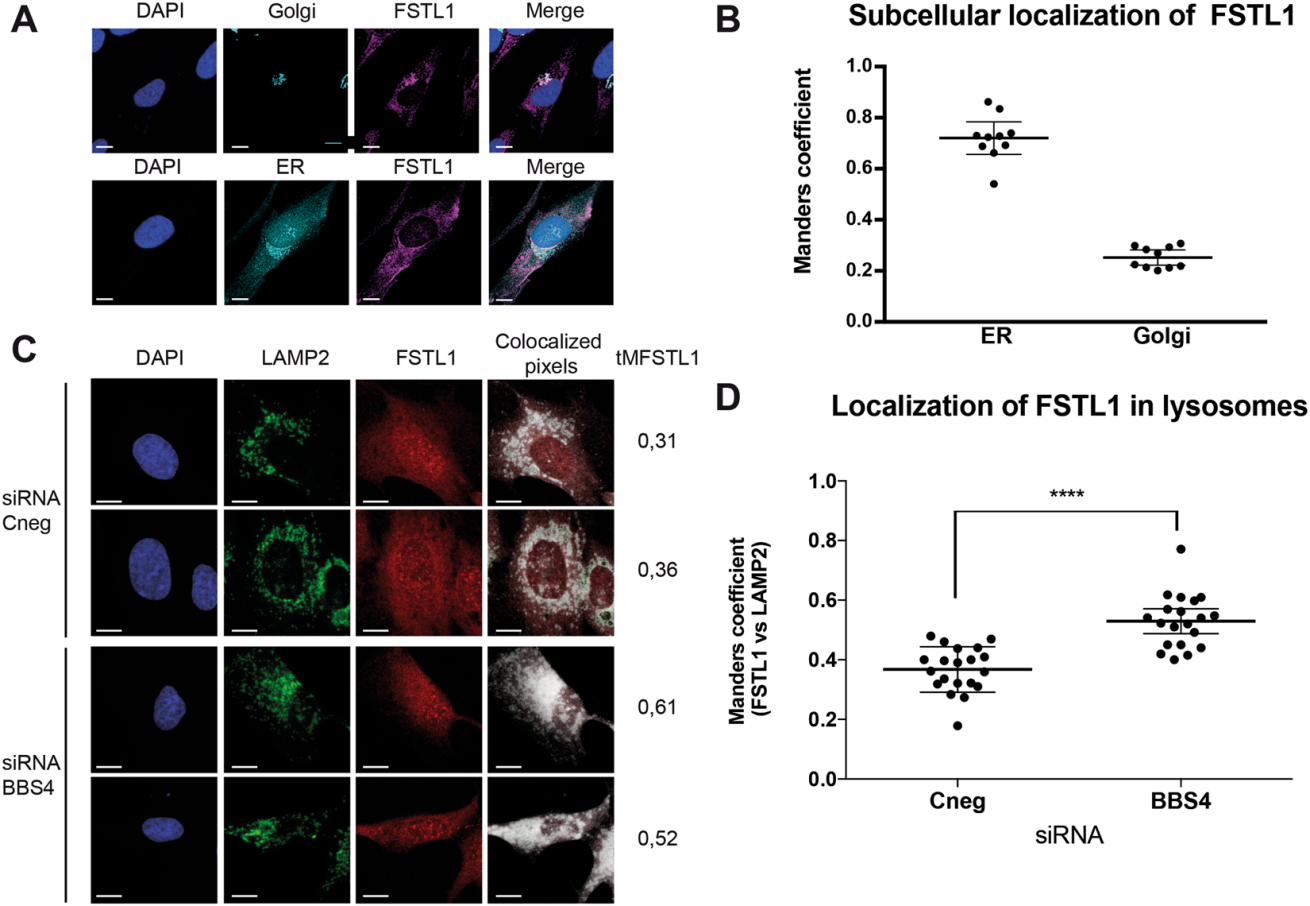
FSTL1 accumulates in lysosomes in the absence of BBS4. **(A**) Control hTERT-RPE1 were co-immunostained for Golgi (top row, Golgin 97 in *cyan*) or ER (bottom row, Calnexin, *cyan*) together with FSTL1 (*magenta*). **(B)** Analysis of colocalization of FSTL1 with each organelle marker represented as Manders coefficients showing that FSTL1 colocalizes with both organelles. **(C)** hTERT-RPE1 control and BBS4-KD cells were co-immunostained to study colocalization of FSTL1 (*red*) and lysosomes (LAMP2 in *green*). Colocalization is evidenced by colocalized pixels in *white*. **(D)** Manders coefficients were determined showing that in BBS4 KD cells, FSTL1 is accumulated in lysosomes. In all cases, scale bars represent 10 µm and ****: *P* < 0,0001, t-test.

FSTL1 is a secreted protein that presumably uses the constitutive secretory pathway through the endoplasmic reticulum and Golgi apparatus. In agreement with previous reports^37^, our confocal microscopy analysis showed a wide cytoplasmic distribution of FSTL1 in punctate structures consistent with intracellular vesicles (Fig. 3). Given the diffuse nature of the FSTL1 localization pattern, we calculated Manders co-localization coefficients^45, 46^. FSTL1 colocalized with Calnexin (ER), Golgin 97 (Golgi) and LAMP2 (lysosomes; Fig. 3A–D). Upon BBS4 KD, we did not observe consistent changes of FSTL1 colocalization with ER or Golgi markers (not shown). In contrast, the depletion of BBS4 resulted in a significant increase of the co-localization coefficients of FSTL1 with LAMP2 from M = 0,36 to M = 0,53 (Fig. 3C,D; *P* < 0.0001). Therefore, these data reinforce our previous biochemical results indicating that in BBS4 KD cells FSTL1 accumulates in lysosomes.

### Secreted FSTL1 plays a role in ciliogenesis

**Figure 4:**
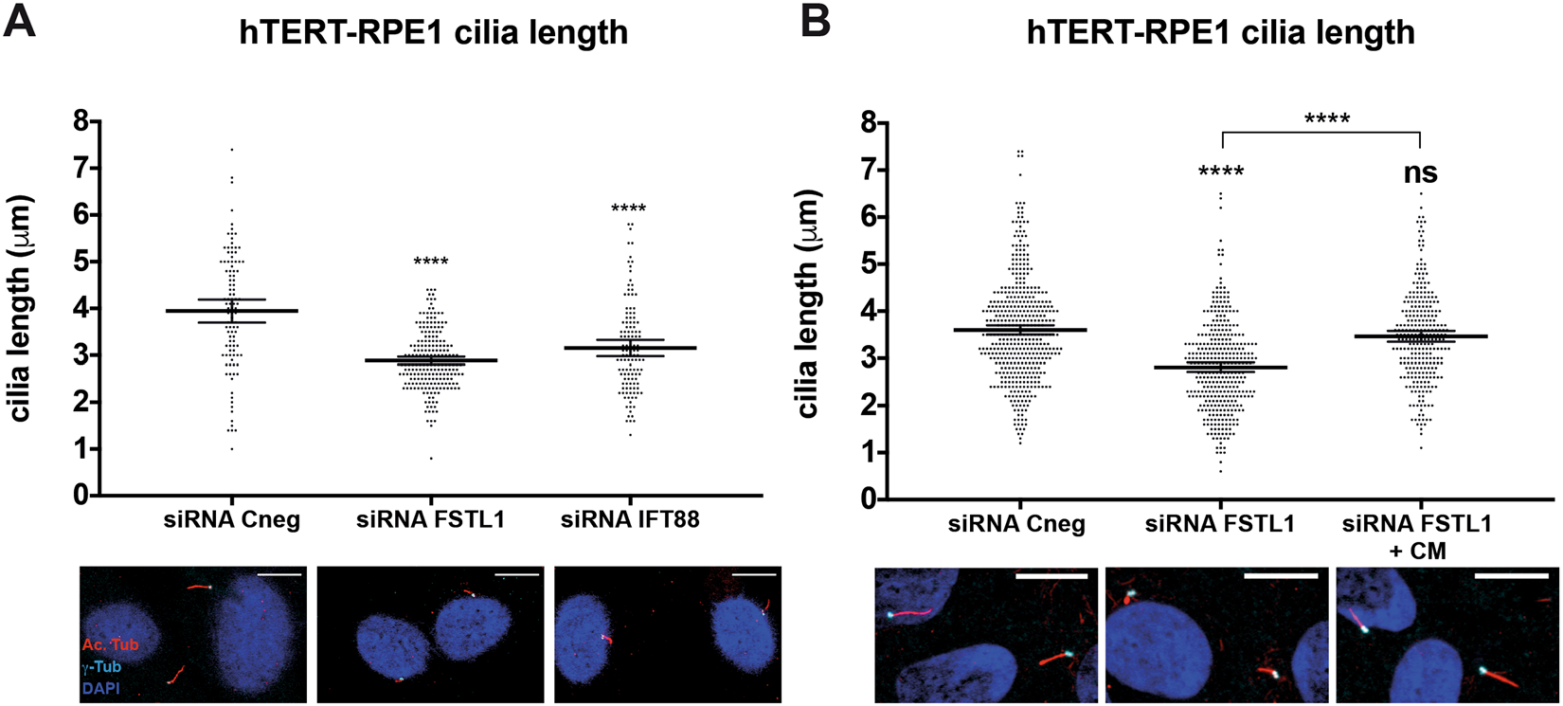
FSTL1 regulates ciliogenesis. (**A**) Upper panels: primary cilia length was measured in hTERT-RPE1 cells. FSTL1 KD cells present shorter cilia than control cells and comparable to IFT88 KD cells. Scatter plots with a line at the mean are shown and error bars represent 95% confidence intervals. Lower panels: micrographs showing basal bodies (γ-tubulin) in *cyan*, cilia (acetylated tubulin) in *red* and nuclei in *blue* (DAPI). The results shown are representative of four independent experiments. **(B)** Upper panel: hTERT-RPE1 FSTL1 KD cells were incubated with conditioned media starting at 24 hours after silencing of FSTL1 and primary cilia length was measured after 24 additional hours. In cells incubated with conditioned media, cilia length was comparable to that of control cells. Scatter plots with a line at the mean are shown and error bars represent 95% confidence intervals. The results shown are representative of two independent experiments. Lower panels: micrographs showing basal bodies (γ-tubulin) in *cyan*, cilia (acetylated tubulin) are in *red* and nuclei are in *blue* (DAPI). Statistical significance is shown as compared to siRNA Cneg except when noted. In all cases, scale bars represent 10 µm. ns: P > 0.05; and ****: *P* < 0,0001, ANOVA test.

Given the known link between the BBS proteins and cilia, we next tested whether FSTL1 plays a role in ciliogenesis. We measured cilia length in FSTL1 KD hTERT-RPE1 cells using immunofluorescence and confocal microscopy. Cilia in FSTL1 KD cells were significantly shorter than in control cells, presenting means of 2.9 µm and 3.9 µm respectively (*P* ≤ 0.0001; Fig. 4A). Cilia were affected similarly when IFT88 was knocked down (Fig. 4A). Since FSTL1 is a secreted protein we next investigated whether the intracellular protein is important for ciliogenesis or if this activity is achieved by secreted FSTL1. We collected conditioned media containing FSTL1 from 24 hours hTERT-RPE1 cell cultures. FSTL1 KD cells incubated with conditioned media showed a recovery in cilia length (3.5 µm) suggesting that the FSTL1 secreted to the media rescues the effect of FSTL1 knockdown (Fig. 4B).

### FSTL1 levels correlate with BBS4 and cilia during differentiation of 3T3-L1 pre-adipocytes

The BBS proteins, the primary cilium, and FSTL1 have been linked to the process of adipogenesis due to their role in pre-adipocyte differentiation. The expression of BBS genes and the presence of cilia are dynamic throughout differentiation^30, 31^. BBS4 knock-down has been reported to increase cell proliferation and fat accumulation in 3T3-F422A cells^32^, and FSTL1 has been proposed to be a marker of preadipocytes given that its levels drop along differentiation into adipocytes^35^. Importantly, the role of FSTL1 in the process has not been established. Therefore, to continue characterizing the functional interaction between Bbs4, cilia and Fstl1, we turned to 3T3-L1 pre-adipocytes.

**Figure 5:**
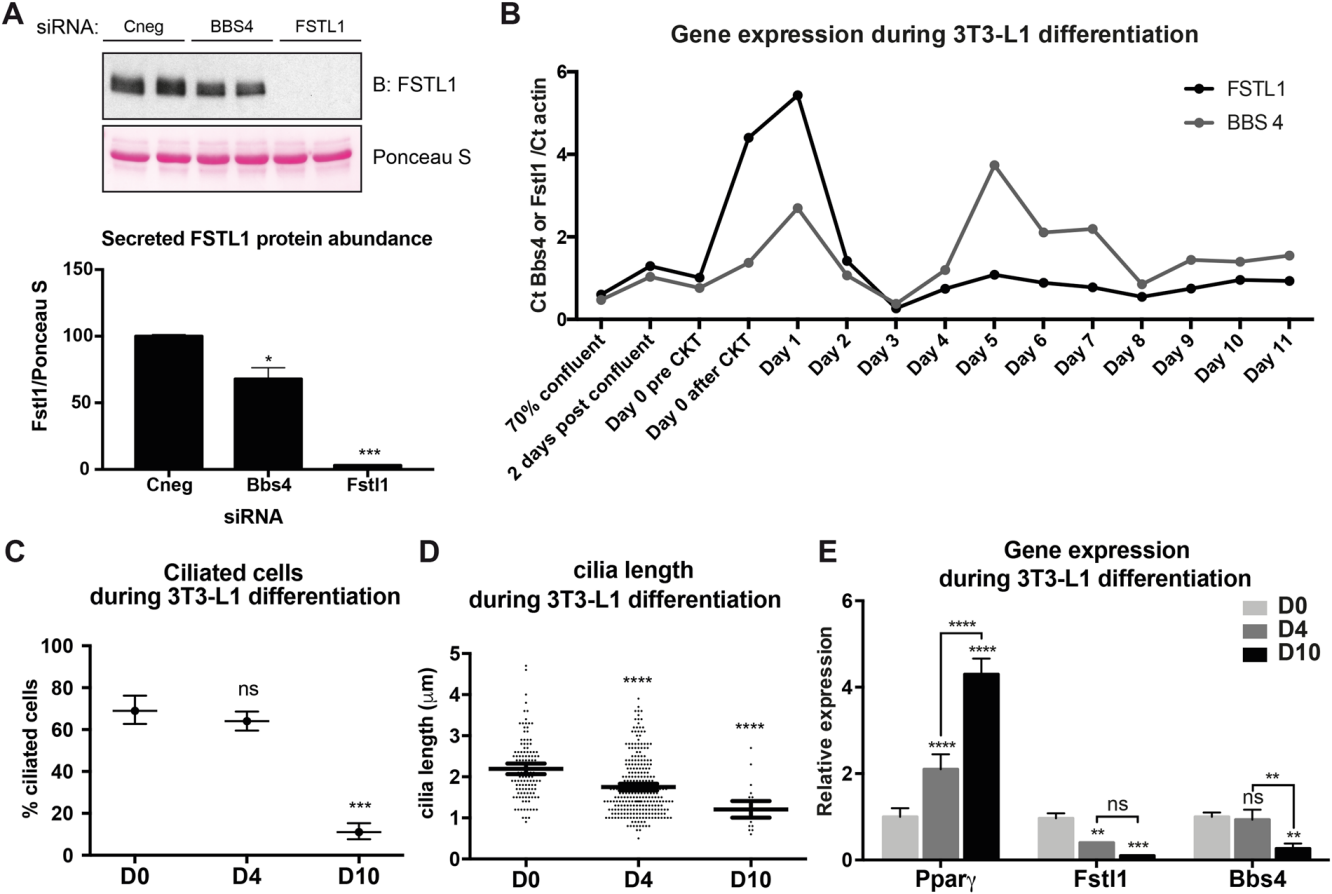
Cilia, BBS4 and FSTL1 are coordinated during 3T3-L1 differentiation. **(A)** 3T3-L1 cells were transfected with control siRNA or siRNA Bbs4 and secreted Fstl1 was detected in the supernatant by Western blot anti-Fstl1 (upper panel). Densitometry analysis of western blot normalized with Ponceau-S staining of an unspecific blotted protein as a loading control (lower panel). Knocking-down Bbs4 also reduces Fstl1 secretion in 3T3-L1 cells. Full-length blot and gel are shown in Figure S9A. **(B)** qRT-PCR analysis of *Fstl1* and *Bbs4* genes expression in 3T3-L1 cells during differentiation from proliferative cells to day 11 after induction to differentiate. Error bars represent standard deviation. Three biological replicates were analyzed. **(C)** The percentage of ciliated cells was analyzed in 3T3-L1 cells at different time points during differentiation: day 0, day 4 and day 10. Mean values are shown and error bars represent 95% confidence intervals for the mean. **(D)** Cilia length was analyzed in 3T3-L1 cells at the same time points. Data is shown as scatter plots with a line at the mean and error bars representing 95% confidence intervals. **(E)** mRNA levels of the adipocyte differentiation marker *Pparγ*, *Fstl1* and *Bbs4* were analyzed in 3T3-L1 cells at the same time points during differentiation. The results shown are representative of multiple independent experiments (see also controls in Figs 6 and 7). Statistical significance is shown as compared to D0 except when noted and ns: P > 0.05; *: *P* = 0,01-0,05; **: *P* = 0,001–0,01; ***: *P* = 0,0001–0,001 and ****: *P* < 0,0001, hypothesis test for proportions, ANOVA or Kruskal Wallis test.

**Figure 6:**
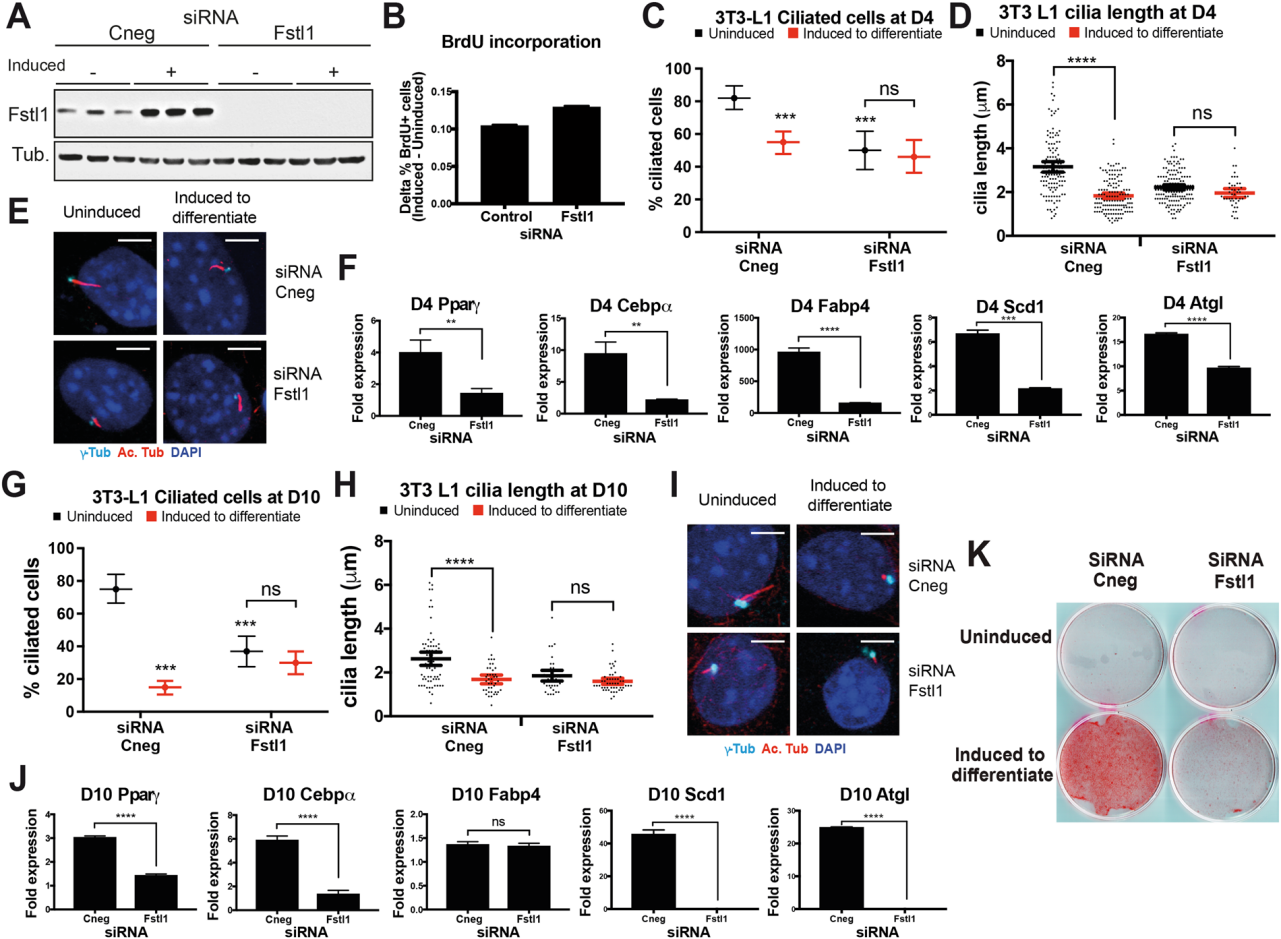
Blocking Fstl1-mediated ciliogenesis inhibits 3T3-L1 preadipocyte differentiation. **(A**) Western blot of Fstl1 showing upregulation of the protein upon induction to differentiate and the levels of the protein in Fstl1 KD 3T3-L1 cells. The data is representative of two independent experiments. Full-length blots are shown in Figure S9B. (**B**) Clonal expansion in 3T3-L1 cells upon induction to differentiate was measured by a pulse of BrdU and immunofluorescence. The difference in the percentage of BrdU positive cells between induced an uninduced cultures is shown. Fstl1 KD does not inhibit clonal expansion. Result of two independent experiments with three biological replicates each. Approximately 500 cells were counted per condition, per experiment. **(C** and **G)** The percentage of ciliated cells was analyzed in 3T3-L1 cells at day 4 (C) and day 10 (G) of differentiation. Data are shown as a line at the mean and error bars represent 95% confidence intervals. **(D** and **H)** Cilia length was measured in 3T3-L1 cells at day 4 (D) and day 10 (H). Approximately 100 cilia were measured per condition, per experiment. Scatter plots with a line at the mean are shown and error bars represent 95% confidence intervals. **(E** and **I**) Micrographs showing examples of cilia used in the analysis at D4 and D10 respectively. Basal bodies (γ-tubulin) are in *cyan*, cilia (acetylated tubulin) are in *red* and nuclei (DAPI) are in *blue*. Scale bars represent 5μm. **(F** and **J**) qRT-PCR analysis of the adipocyte differentiation markers *Pparγ*, *Cebpα*, *Fabp4*, *Scd1* and *Atgl* in 3T3-L1 cells at day 4 (F) and day 10 (J) of differentiation. Bars represent the fold change upon induction of each gene relative to *Gapdh* comparing induced with uninduced cells. Error bars represent standard deviation. (**K**) Oil red staining comparing lipid accumulation between control and Fstl1 KD cells. The data shown are representative of two independent experiments. ns: P > 0.05; **P = 0,001-0,01; ***P = 0,0001–0,001 and ****P < 0,0001, hypothesis test for proportions, Kruskal Wallis or t-test.

First, we confirmed that depletion of Bbs4 in 3T3-L1 cells also resulted in Fstl1 secretion defects, showing a 40% reduction (Fig. 5A). Next, we examined the expression patterns of *Bbs4* and *Fstl1* during the differentiation of 3T3-L1 cells. Cells were collected at different time points: at approximately 70% confluency; 2 days after reaching 100% confluency; and daily after differentiation day 0 (when differentiation media was added) up to day 11. In agreement with reported data^30^, using qRT-PCR we observed that *Bbs4* was upregulated transiently upon addition of differentiation medium. Bbs4 expression came down around day 2 post-differentiation and presented a second peak of expression at day 5 (Fig. 5B). *Fstl1* gene expression also followed a biphasic curve peaking shortly after the onset of differentiation (5.3-fold relative to day 0), dropping by day 4, and remaining low thereafter (Fig. 5B). We next examined the presence and length of primary cilia at days 0, 4 and 10 after induction of differentiation (D0, D4 and D10) (Fig. 5C,D). Consistent with previous reports^31, 47^, we found that 69% of cells at day D0 had primary cilia, which remained present until D4 at which point 64% of cells were still ciliated. In contrast, only 11% of cells had cilia at D10 (Fig. 5C). Cilia length decreased from D0 (2.2 µm) to D4 (1.8 µm) to D10 (1.6 µm) (Fig. 5D), compared to uninduced control cells in which cilia length ranged from 2.7 µm to 3.4 µm on average at D4 and 2.4 µm to 3.0 µm at D10 (Fig. 6D and H, 7C). We followed *Pparγ* expression, a marker of adipocyte differentiation^48^, which increased 2-fold at D4 and 4-fold at D10, while *Fstl1* and *Bbs4* expression dropped significantly (Fig. 5E). The differentiation markers *Cebpα* and *Fabp4* ^48^ were also upregulated during differentiation (Fig. S4). Moreover, Fstl1 levels were reduced significantly upon induction of obesity in C57/BL6 mice fed *ad libitum* with a Western diet during 10 weeks (Fig. S5).

### Knocking-down *Fstl1* in 3T3-L1 preadipocytes impairs differentiation

Overall our data show that BBS4 regulates FSTL1 and we hypothesized that this finding could be relevant to understand the previously reported role of BBS4 in adipocyte differentiation. As mentioned, FSTL1 can be considered a preadipocyte marker given that it is downregulated as adipocyte differentiation progresses^35^, but whether the protein plays an active role in the process was not determined. We therefore tested whether modulating FSTL1 levels could affect 3T3-L1 differentiation. Since *Fstl1* presents an initial phase with high expression and a second phase where its level drops (Fig. 5), we hypothesized that Fstl1 could have a dual effect: it might be required at the onset of differentiation while it needs to be downregulated once cells engage in the differentiation process.

To test the requirement for Fstl1 at the beginning of differentiation, we knocked it down in 3T3-L1 preadipocytes before adding differentiation media thus reducing both Fstl1 basal levels and its peak in expression associated with the induction to differentiate (Fig. 6A). After induction 3T3-L1 cells present a clonal expansion phase^49^ that was not impaired by Fstl1 KD as evidenced by BrdU incorporation (Fig. 6B). We then sampled control and Fstl1 KD cells at D4 and D10 and examined i) the percentage of ciliated cells, ii) cilia length and iii) the expression of the adipogenic transcription factors *Pparγ*, *Cebpα, Fabp4, Scd1* and *Atgl*. By D4, uninduced Fstl1 KD cells had fewer and shorter cilia than control cells (50% vs 82% and 2.2 µm vs 3.1 µm; Fig. 6C–E). Furthermore, the induction to differentiate in Fstl1 KD cells did not result in a further drop in the number (Fig. 6C) or length of cilia (Fig. 6D): cilia length in induced control cells was 1.8 µm compared to 3.1 µm in uninduced cells, whereas in Fstl1 KD cells cilia length was not significantly different (2.2 µm in uninduced and 1.9 µm in induced cells; Fig. 6D–E; Suppl. Table 1). Therefore, Fstl1 plays a role at the beginning of differentiation favoring ciliation and ciliary length. In addition, Fstl1 KD impaired the upregulation of adipogenic markers upon induction to differentiate (Fig. 6F).

At D10, control cells induced to differentiate exhibited significantly fewer cilia than uninduced cells (15% compared to 74%). Similar to D4, Fstl1 KD cells presented fewer cilia and this percentage was not reduced further by the stimulus to differentiate (37% ciliation vs 30% respectively; Fig. 6G). In control cells and after 10 days of differentiation, the rare primary cilia that we were able to measure were shorter in induced cells compared to uninduced cells (1.7 µm long compared to 2.4 µm). In contrast, cilia in differentiating Fstl1 KD cells were not significantly different in length between induced (1.5 µm) and uninduced (1.8 µm) cells (Fig. 6H–I, Suppl. Table 1). The expression of *Pparγ*, *Cebpα*, *Scd1* and *Atgl* was significantly reduced in Fstl1 KD cells at D10 whereas *Fabp4* was comparable to controls (Fig. 6J). Finally, staining cells at D10 with the oil-soluble dye Oil Red O showed that knockdown of Fstl1 impaired lipid accumulation (Fig. 6K). Thus, our results show that Fstl1 depletion at the onset of differentiation significantly affects cilia and alters adipogenesis in 3T3-L1 cells.

### Maintaining high Fstl1 levels interferes with the differentiation of 3T3-L1

Finally, we tested whether down-regulation of *Fstl1* is also a required event during differentiation. We first attempted to maintain Fstl1 levels up by using conditioned media from D0 onwards: conditioned media was obtained from 3T3-L1 cultures and media collected from Fstl1 KD cells was used as control (Fig. S6A). Along the entire differentiation protocol the addition of conditioned media resulted in a mild effect on cilia: by D4 it did not affect the percentage of ciliated cells nor its reduction upon induction to differentiate. The expected cilia shortening triggered during differentiation was observed with both media, albeit it was milder in cells cultured in conditioned media (Suppl. Table 2, Fig. S6B,C). At D10 we observed a subtle effect on both the reduction in the percentage of ciliated cells and cilia shortening (Suppl. Table 2). Similarly, the expression of adipogenic markers was variably affected. While *Pparγ* expression was upregulated at D4 in the presence of conditioned media, *Cebpα* and *Fabp4* were not affected (Fig. S6D). By day 10, the conditioned media with Fstl1 inhibited the upregulation of *Pparγ*, did not affect the expression of *Cebpα* and *Fabp4* (Fig. S6E) and resulted in a modest effect on lipid accumulation (Fig. S6F). Thus, our results suggested a mild perturbation of differentiation if Fstl1 levels are maintained high.

**Figure 7:**
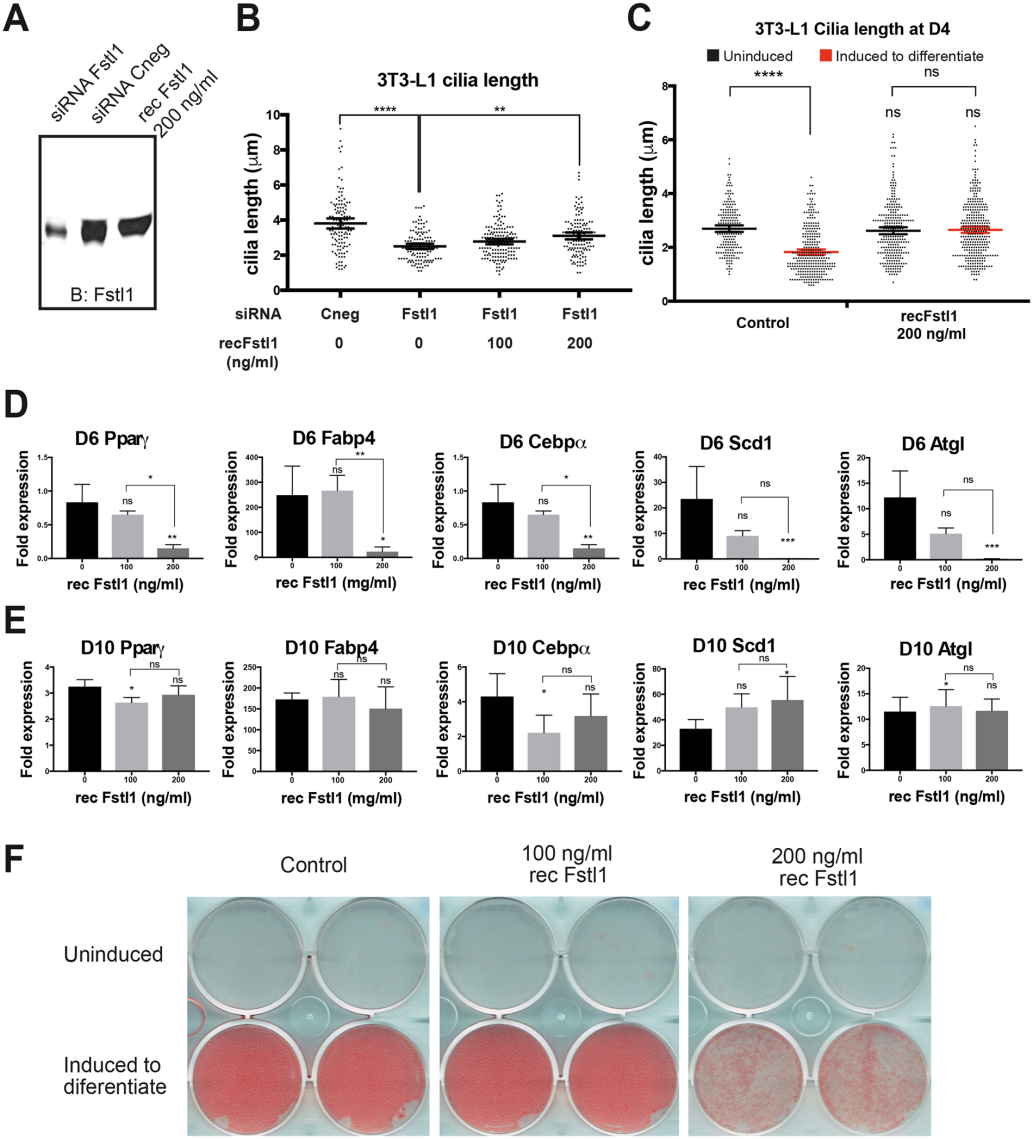
Addition of recombinant Fstl1 interferes with 3T3-L1 preadipocyte differentiation. (**A**) Western blot showing comparable levels of Fstl1 in supernatant from control cells and recombinant Fstl1 diluted in fresh culture media at 200 ng/ml. **(B)** Cilia length was measured in control and Fstl1 KD 3T3-L1 cells supplemented with 0, 100 ng/ml or 200 ng/ml of recombinant Fstl1. Cilia length was rescued in a dose dependent manner by the addition of Fstl1. Data are shown as a line at the mean and error bars represent 95% confidence intervals. At least 120 cilia were measured per condition and results shown are representative of three independent experiments. **(C)** Cilia length at D4 was measured in uninduced and induced to differentiate control cells and cells cultured with 200 ng/ml of recombinant Fstl1. Scatter plots with a line at the mean are shown and error bars represent 95% confidence intervals. (**D**,**E**) qRT-PCR analysis of gene expression of differentiation markers *Pparγ*, *Cebpα*, *Fabp4*, *Scd1* and *Atgl* in 3T3-L1 cells at day 6 (**D**) and day 10 (**E**) of differentiation. Bars represent the fold change upon induction of each gene relative to *Gapdh* comparing induced cells with uninduced cells. Error bars represent standard deviation. Two biological and three technical replicates were analyzed. (**F**) Oil red staining showing lipid accumulation in induced control cells in comparison with cells supplemented with recombinant Fstl1 protein. ns: P > 0.05; *: P = 0,01-0,05; **: P = 0,001-0,01; ***: P = 0,0001–0,001 and ****: P < 0,0001, ANOVA, or t-test.

To further evaluate this possibility we used purified recombinant Fstl1. We first tested whether the recombinant protein was functional by assessing its capacity to rescue the cilia phenotype of Fstl1 KD 3T3-L1 cells. We knocked down Fstl1 and added Fstl1 protein 24 hours after transfecting with the siRNA oligo: 200 ng/ml of protein to reach levels comparable to secreted protein (Fig. 7A), or 100 ng/ml as a suboptimal dose. Cilia length was rescued in a dose-response manner increasing from 2.5 µm in Fstl1 KD cells to 2.8 and 3.1 µm with 100 and 200 ng/ml of recombinant Fstl1 respectively (Fig. 7B). Thus, our results showed that the recombinant protein was functional, at least regarding its role in cilia, and further supported our previous observation that secreted Fstl1 plays a role in ciliogenesis.

We then evaluated the effect of supplementing the media with 200 ng/ml of Fstl1 recombinant protein during 3T3-L1 cell differentiation by assessing cilia at D4, adipogenic markers at D6 and D10, and lipid accumulation at D10. At D4, the addition of Fstl1 protein completely blocked the cilia shortening that is triggered with the addition of differentiation media: in control cells cilia changed from 2.7 µm to 1.9 µm upon induction while cilia in cells cultured with 200 ng/ml of Fstl1 were 2.7 µm long both in the uninduced and induced conditions (Fig. 7C, Suppl. Table 3). This in turn correlated with a reduction in the levels of *Pparγ*, *Cebpα*, *Fabp4*, *Scd1*, and *Atgl* (Fig. 7D). Interestingly however, by day 10, despite a marked reduction in lipid accumulation in cells treated with 200 ng/ml Fstl1 (Fig. 7F), the expression of the different adipogenic markers was comparable to controls (Fig. 7E). Therefore, maintaining Fstl1 levels affected differentiation likely delaying it. Collectively, our data suggest that both the initial high levels of Fstl1 as well as its reduction during differentiation are important steps during the differentiation of 3T3-L1 cells.

## Discussion

Despite having a common cellular defect, the ciliopathies present overlapping but not identical phenotypes. In addition, the same clinical manifestation can vary in its presentation among different ciliopathies. This variability can be explained by the specific gene affected, the type of mutation, the specific role of the encoded protein in the context of the cilium, and importantly, extra-ciliary functions that the protein might have. Here we show that BBS4 regulates the function of FSTL1 through a dual mechanism, cilia dependent and independent, at the mRNA level and the secretion of the protein respectively.

Our data indicate that reducing BBS4 results in decreased *FSTL1* mRNA levels. Given that BBS4 plays a role in cilia, through its role in the BBSome^17^, this is likely a cilia-dependent effect. Supporting this possibility, targeting *IFT88* also downregulated *FSTL1* mRNA. Furthermore, *FSTL1* was originally identified as a TGFβ1 target and this signaling pathway is regulated by the cilium with TGFβ receptors localizing in the ciliary compartment and ciliary pocket at the base of the organelle^33, 50^.

In addition, we show that BBS4 regulates the cellular trafficking of FSTL1 in a cilia-independent manner. Depletion of BBS4 resulted in the accumulation and degradation of FSTL1 in lysosomes. Consistently, intracellular levels of FSTL1 were rescued by lysosome inhibition, which did not rescue the secretion of FSTL1 suggesting that inhibiting protein degradation did not fix the underlying intracellular trafficking defect caused by BBS4 KD. Importantly, chloroquine treatment did not rescue the FSTL1 reduction in IFT88 KD cells supporting the interpretation that, in this latter case, FSTL1 abundance is regulated exclusively at the transcriptional level. Based on the reported involvement of BBS4 in vesicle sorting and trafficking, together with our lysosomal degradation data, we propose that the depletion of BBS4 results in a shift of FSTL1 from the secretory to the endo-lysosomal degradation pathway. Furthermore, BBS2 knockdown also led to a FSTL1 secretion defect suggesting that this function of BBS proteins could occur in the context of the BBSome, a possibility that will have to be studied further.

These results raise two main questions: i) what is the mechanism by which BBS4 regulates FSTL1 secretion? and ii) could a FSTL1 secretion defect contribute to the etiology of BBS-associated phenotypes? Regarding the mechanism, it is known that the BBS proteins, through the activity of the BBSome, direct Golgi-derived vesicles to the base of the primary cilium by recognizing ciliary membrane proteins^15, 17, 51^ and we have shown that BBS4 participates in recycling the Notch receptor to the plasma membrane^26^. Interestingly, recycling endosomes act as sorting checkpoints, coordinating trafficking pathways in close proximity to the basal body^52^. Therefore, BBS4, in the context of the BBSome, might direct FSTL1-containing vesicles towards the plasma membrane. However, FSTL1 is a secreted protein expected to be present in the lumen of the Golgi compartment and post-Golgi vesicles whereas the BBS proteins are cytosolic. Although this work began by finding a putative physical interaction between BBS4 and FSTL1, further studies will be needed to first confirm this interaction in a physiological setting and then dissect when and where does it occur inside the cell. One possibility is that a pool of FSTL1 might be secreted by a non-classical route depending on the BBS proteins, analogous to FGF2 which is secreted by direct translocation across the plasma membrane^53^. However, FSTL1 presents a typical ER signal peptide and is N-glycosylated, a post-translational modification that occurs in the ER and Golgi^54^. Therefore, the mechanism by which BBS proteins affect FSTL1 secretion is unknown and understanding it will be critical to completely elucidate the role of BBS proteins in intracellular traffic. Regarding the second question, our results strongly document a FSTL1 defect upon depletion of BBS4 thus raising the intriguing possibility that this could contribute to the pathogenesis of BBS phenotypes. Here we studied the role of FSTL1 in the differentiation of 3T3-L1 cells as a first approximation to test this hypothesis.

Previous reports have linked the BBS proteins, cilia and FSTL1 with adipocyte differentiation. Studies of gene expression dynamics of *Bbs*1-4, 6-9 and 11 in the murine cell line 3T3-F422A^30^, and *BBS10* and *BBS12* in human mesenchymal stem cells (hMSC)^31, 55^ showed maximum values for *BBS* transcripts between D2 and D4 of differentiation. Transient ciliogenesis is observed during adipogenesis of human adipose stem cells, followed by a decrease in cilia number and length in mature adipocytes^47^. In 3T3-L1 cells, blocking ciliogenesis by suppressing *Ift88* inhibits differentiation^56^. During pre-adipocyte to adipocyte differentiation FSTL1 expression shows a transient increase during the first two days followed by a dramatic decrease^35^. However, whether FSTL1 plays an active role during adipogenesis was not known. Here we assessed the role of Fstl1 during 3T3-L1 differentiation hypothesizing that the initial phase of increase and the phase of decrease are both important, affecting the initiation and progression of differentiation respectively. Thus, we used two different approaches to modulate Fstl1 abundance: i) we knocked down *Fstl1* expression prior to differentiation and ii) we impaired Fstl1 down-regulation using a differentiation cocktail prepared with conditioned media containing Fstl1 or by adding purified recombinant Fstl1 directly. Our results indicate that both phases are relevant for 3T3L1 differentiation.

Our understanding of how FSTL1 modulates 3T3-L1 differentiation is still incomplete. While we cannot discard a role for Fstl1 in cell proliferation, Fstl1 KD did not affect clonal expansion of 3T3-L1 upon induction to differentiate. In contrast, our data do highlight a complex interplay between the BBS proteins, FSTL1 and cilia. First, *Bbs* and *Fstl1* gene expression and the presence of cilia correlate during differentiation. In agreement with previous reports, our data show that *Bbs4* and *Fstl1* are synchronized and transiently upregulated shortly after the onset of differentiation, while cilia are also transiently observed and later reabsorbed as differentiation progresses. Second, our results unveil a link between the BBS proteins and cilia with FSTL1, which in turn we report here as a novel regulator of ciliogenesis, both in hTERT-RPE and 3T3-L1 cells. Accordingly, reducing Fstl1 levels from the start of differentiation resulted in shorter and fewer cilia by day 4 and impaired differentiation. Importantly, maintaining Fstl1 levels high also perturbed cilia dynamics by inhibiting their reabsorption and affected differentiation, likely delaying it. Collectively, these results suggest that *Fstl1* expression in pre-adipocytes is required for transient ciliogenesis and commitment to the differentiation program. In addition, our results point to a cilia-Fstl1 feedback loop, whereby cilia modulates Fstl1 expression and Fstl1 participates in ciliogenesis. Defining the exact order of events will require further work but our results indicate that Fstl1 downregulation contributes to cilia reabsorption and differentiation in 3T3-L1 cells.

Therefore, Fstl1 could modulate, at least indirectly, cilia-mediated signaling pathways important for adipogenesis such as Shh and Wnt. Shh signaling has been shown to influence the balance between the osteoblastic and adipogenic pathways^57–60^. Also, in 3T3L1 cells, Hedgehog signaling was shown to be active in preadipocytes and then decrease during differentiation^61^. Canonical Wnt signaling activation leads to *PPARγ* and *CEBPα* downregulation and inhibition of differentiation^62^. Therefore, the reduction in Fstl1 levels could promote or ensure cilia reabsorption and thus facilitate the downregulation of cilia-dependent anti-adipogenic pathways during differentiation. In addition, FSTL1 has been shown to act as an inhibitor of bone morphogenetic protein 4 (BMP4)^63, 64^, a member of the transforming growth factor β (TGF-β), which in turn is an important player during adipogenesis, driving the commitment of mesenchymal stem cells to the adipogenic lineage and differentiation of preadipocytes^65–69^.

Our data uncover a novel role for FSTL1 regulating ciliogenesis in a non-cell autonomous manner. Complete depletion of Fstl1 in mice results in perinatal death due to severe malformations, particularly affecting lung and skeletal development and dependent on misregulated BMP signaling^63, 64^. It will be interesting to evaluate the status of cilia in this model and to determine whether the role of FSTL1 in ciliogenesis relies on its activity as a BMP4 inhibitor. Also, a receptor for FSTL1, DIP2A, was identified in endothelial cells^70^ and future work will be aimed at determining whether DIP2A mediates the role of FSTL1 in ciliogenesis and adipogenesis.

Understanding the functional relationship between BBS proteins, cilia and FSTL1 will provide critical insight to dissect the cellular basis of BBS. Depletion of Bbs4 promotes proliferation and triglyceride accumulation in murine 3T3-F422A cells^32^ and Bbs4-null mice develop obesity as well as other BBS features such as retinal degeneration^71, 72^. Similarly, cells from BBS10 and BBS12 patients differentiate and accumulate more adipocytes and more triglycerides and secrete more leptin^31, 55^. Some of these results might be difficult to reconcile with the requirement for cilia at the onset of adipocyte differentiation. However, targeting individual BBS proteins does not necessarily inhibit ciliogenesis. For example, *Bbs2*, *Bbs4* and *Bbs6* knockout mice do form cilia^72–74^. Thus, one possibility is that in the absence of proteins such as BBS4, albeit compromised, cilia are functional enough to allow adipocyte differentiation to begin. Then, a lower level of secreted FSTL1 might generate a sensitized background where adipogenesis could be favored. *In vivo* work will be needed to fully evaluate this intriguing possibility. Our results highlight the need to fully understand the role of ciliary proteins both in and outside the cilium, as these more likely specific functions might underlie important differences in the presentation of a given phenotype across ciliopathies.

## Methods

### Reagents, antibodies

Bovine serum albumin, Ponceau S, Sigmafast protease inhibitor cocktail tablets, Rosiglitazone and Insulin from Sigma. Primary antibodies: mouse anti-α tubulin clone B-5-1-2, mouse anti-acetylated tubulin clone 6-11-B-1, rabbit anti-γ tubulin and rabbit anti-laminin from Sigma; goat anti-human FSTL1, goat anti-mouse Fstl1, and mouse Fstl1 recombinant protein from R&D Systems, rabbit anti-BBS4 from ProteinTech, rabbit anti-LC3B from Cell Signaling Technology, mouse anti-human golgin-97 clone CDF4 from Invitrogen, and rabbit anti-calnexin, mouse anti-LAMP2 clone H4B4 and rat anti-BrdU from Abcam. Horseradish peroxidase (HRP)-conjugated secondary antibodies anti-mouse and anti-goat from Santa Cruz and anti-rabbit from Sigma. Alexa Fluor (AF)−594 donkey anti-goat, AF-488 donkey anti-mouse, AF-633 goat anti-rabbit, Alexa Fluor 488-conjugated anti-rat and tetramethylrhodamine goat-anti mouse were from Invitrogen. TRIzol reagent, Lipofectamine RNAiMAX and stealth RNAs were obtained from Invitrogen. Dexamethasone and 3-Isobutyl-1-Methylxanthine (IBMX) were obtained from AppliChem.

### Yeast-two hybrid assay

The Cytotrap cytoplasmic yeast-two hybrid assay (Stratagene) was performed using a human fetal brain library as previously described^75^.

### Cell culture and transfection

hTERT-RPE1 cells were maintained in DMEM-F12 with Hepes with 10% FBS, PS and 0,01 mg/ml Hygromicin (Sigma). 3T3-L1 cells were maintained in DMEM with glutamax, high glucose and high pyruvate with 10% FBS and PS. For siRNA experiments, stealth siRNA duplex ARN oligos were transfected using Lipofectamine RNAiMax (Invitrogen). To collect conditioned media, cells were transfected with the appropriate siRNA (control or *Fstl1*), media was replaced 24 hours post-transfection, and media was collected after additional 24 hours. The media was centrifuged at 800xg for 5 min to eliminate cell debris.

### Cell lysates, supernatants and Western blot

Cells were lysed at 4 °C for 45 minutes using lysis buffer (50 mM Tris-HCl, pH 7,4, 150 mM NaCl, 5 mM EDTA, 1% sodium deoxycholate, 1% Nonidet P-40) supplemented with a protease inhibitor cocktail (Sigma). Whole cell lysates were obtained as the soluble fraction after centrifugation at 12,000xg for 15 minutes. Secreted proteins were collected with the media from serum-starved (24 hours) cultures and centrifuged for 5 minutes at 800xg. Proteins in lysates (30–50 µg) or supernatant (40 µl) were blotted into PVDF membranes and probed with anti-FSTL1 (1/2000) and anti-tubulin (1/2000), or the appropriate antibodies overnight at 4 °C. Horseradish peroxydase (HRP)-conjugated secondary antibodies were used for western blot. The densitometry of bands was performed using FIJI and Graph Pad Student test calculator.

### siRNA sequences

The siRNA oligonucleotide sequences used in this study are available upon request.

### RNA extraction and qRT-PCR

Total RNA was extracted from cells using TRIzol and cDNA was generated using either the Superscript First strand synthesis system for RT-PCR (Invitrogen) or the Fermentas First Strand cDNA transcription kit (Thermo scientific). Target-specific primers for *FSTL1*, *BBS4*, *BBS2*, *IFT88*, *Pparγ*, *Fabp4*, *Cebpα*, *Scd1*, *Atgl*, *Gapdh* and *Cyclophilin* are available upon request. Platinum SYBR Green qPCR superMix-UDG (Invitrogen), SYBR FAST Universal 2X qPCR Master Mix (Kapa) and LightCycler 480 SybrGreen (Roche) were used in different experiments and reactions were performed either on a Real Time Rotor Gene 6000 PCR (Corbett Research), an Eco Real time PCR System (Illumina) or a LightCycler 480 machine (Roche). All samples were run in triplicate and the CT value was normalized to calculate relative expression of each gene. The rate of gene expression (R) was calculated using the ΔΔCt method with *Cyclophilin*, *Gapdh*, or β-actin as reference genes and relative to a control situation (control siRNA or uninduced cells).

### Immunofluorescence and co-localization studies

Cells grown in coverslips were fixed with ice-cold methanol or freshly made PBS containing 4% paraformaldehyde for 10 minutes, permeabilized with PBS containing 0,1% Triton X-100, and blocked with PBS containing 0.02% Tween-20, 10% FBS and 1% BSA. Coverslips were incubated overnight at 4 °C with goat anti-human FSTL1 1/100, rabbit anti-LAMP2 1/1000, mouse anti-Golgin 97 1/100 or anti-calnexin 1/2000 followed by Alexa Fluor 594-conjugated anti-Goat, Alexa Fluor 488-conjugated anti-rabbit or anti-mouse antibodies. Images were acquired using a Leica SP5 confocal microscope under 63X oil immersion 1.4 NA objective, Zoom of 5X and Z sections of 0,123 µm of thickness and acquired with same gain, offsets an laser powers settings. Co-localization was quantified as Manders coefficients using the colocalization threshold and the Jacop plugin, both available with FIJI software. For cilia staining, cells were probed with rabbit anti-γ tubulin 1/1000, mouse anti-acetylated tubulin 1/1000, and Alexa Fluor 633-conjugated anti-rabbit, Alexa Fluor 488-conjugated anti-mouse antibodies and DAPI. For anti-BrdU staining, cells were permeabilized with PBS containing 2 M HCl and 0.1% tween-20 for 15 minutes at 37 °C, blocked with PBS containing 2% BSA, 0.3% Triton X-100, 150 mM glycine for 1 hour, incubated with rat anti-BrdU 1/100 overnight at 4 °C followed by Alexa Fluor 488-conjugated anti-rat 1/1000 and DAPI.

### 3T3-L1 preadipocyte differentiation

3T3-L1 preadipocytes were maintained as a subconfluent monolayer culture and differentiation was induced by placing cells in differentiation cocktail (DMEM with 10% FBS, PS, 1 μg/ml bovine insulin, 0,25 mM dexamethasone, 0.5 mM IBMX and 2,5 mM Rosiglitazone), for 48 hours and then changing the medium every two days with DMEM with 10% FBS, PS, 1 μg/ml insulin. For the BrdU incorporation assay, cells were grown in coverslips and 10 μM BrdU was added to the differentiation cocktail at the time of induction for 24 hours. Cells were fixed with PBS containing 4% paraformaldehyde for 10 minutes and probed with anti-BrdU and DAPI.

### Statistical data analysis

For cilia length measurements, cilia were measured using the freehand ROI selection tool in FIJI. For all data processing, values were analyzed using GraphPad Prism 7 One-way ANOVA with Tukey´s post hoc test or Student t-test and for data with no normal distribution we used the non-parametrics test Kruskal Wallis. For percentage of ciliated cells and BrdU incorporation assays, the fractions of cells with cilia or BrdU positive cells vs the total number of cells were calculated. Comparison between different experimental conditions was done using a test of Hypothesis specific for comparison of two proportions (hypothesis test for proportions).

In all cases, differences were considered significant when *P* values were smaller than 0,05.

## Electronic supplementary material

Supplementary Information
