# Supplementary material for: BBS4 regulates the expression and secretion of FSTL1, a protein that participates in ciliogenesis and the differentiation of 3T3-L1
Source: Sci Rep. 2017 Aug 29;7:9765. doi: 10.1038/s41598-017-10330-0 (PMC5575278; doi:10.1038/s41598-017-10330-0)
Supplement: Supplementary file 1 — Supplementary Information [file 41598_2017_10330_MOESM1_ESM.pdf]

Supplementary Information

BBS4 regulates the expression and secretion of FSTL1, a protein that participates in ciliogenesis and the differentiation of 3T3-L1

Victoria Prieto-Echagüe[1,2], Sukanya Lodh[3], Laura Colman[2,4], Natalia Bobba[2,4], Leonardo Santos[2,4], Nicholas Katsanis[5], Carlos Escande[2,4], Norann A. Zaghloul[3] and Jose L. Badano[1,2,*]

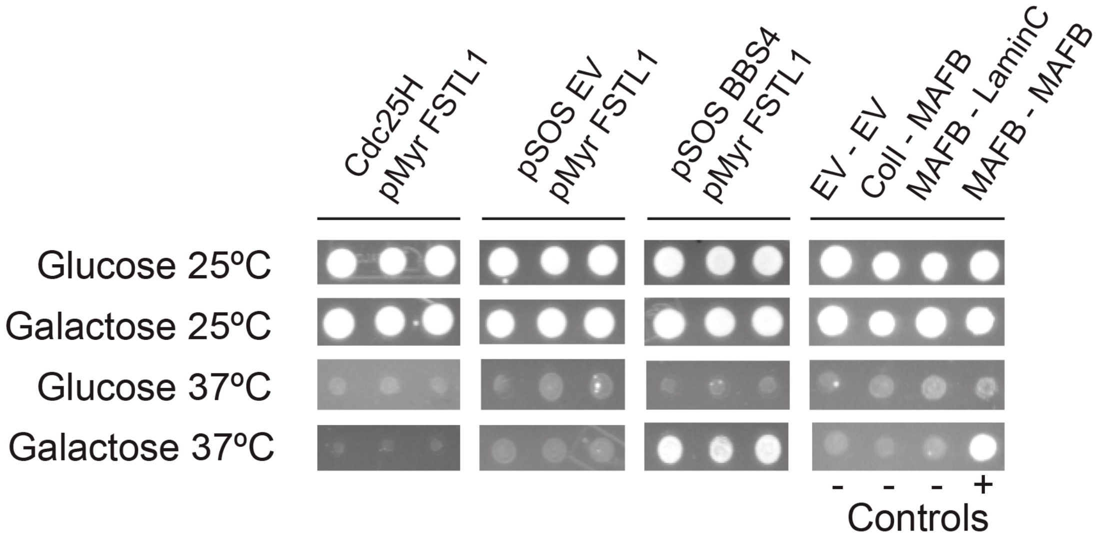

Figure S1: BBS4 and FSTL1 interact in a cytoplasmic yeast two-hybrid screen.
We used a cytoplasmic two-hybrid screen in which a temperature sensitive strain of yeast (cdc25h) is able to grow at 37°C only when the proteins expressed from the pSOS (bait) and pMyr (prey) constructs interact. After the initial screen we tested the interaction between BBS4 and FSTL1 by transforming cdc25h, cdc25h-pSOS EV and cdc25h-pSOS-BBS4 yeasts together with the pMyr FSTL1 construct: only yeast carrying both pSOS-BBS4 and pMyr-FSTL1 were able to grow at 37°C and when cultured on galactose which drives the expression of the Myr-fussion. Controls: positive, MAFB-MAFB; negatives, EV-EV (empty vectors), CollI-MAFB, MAFB-Lamin C.

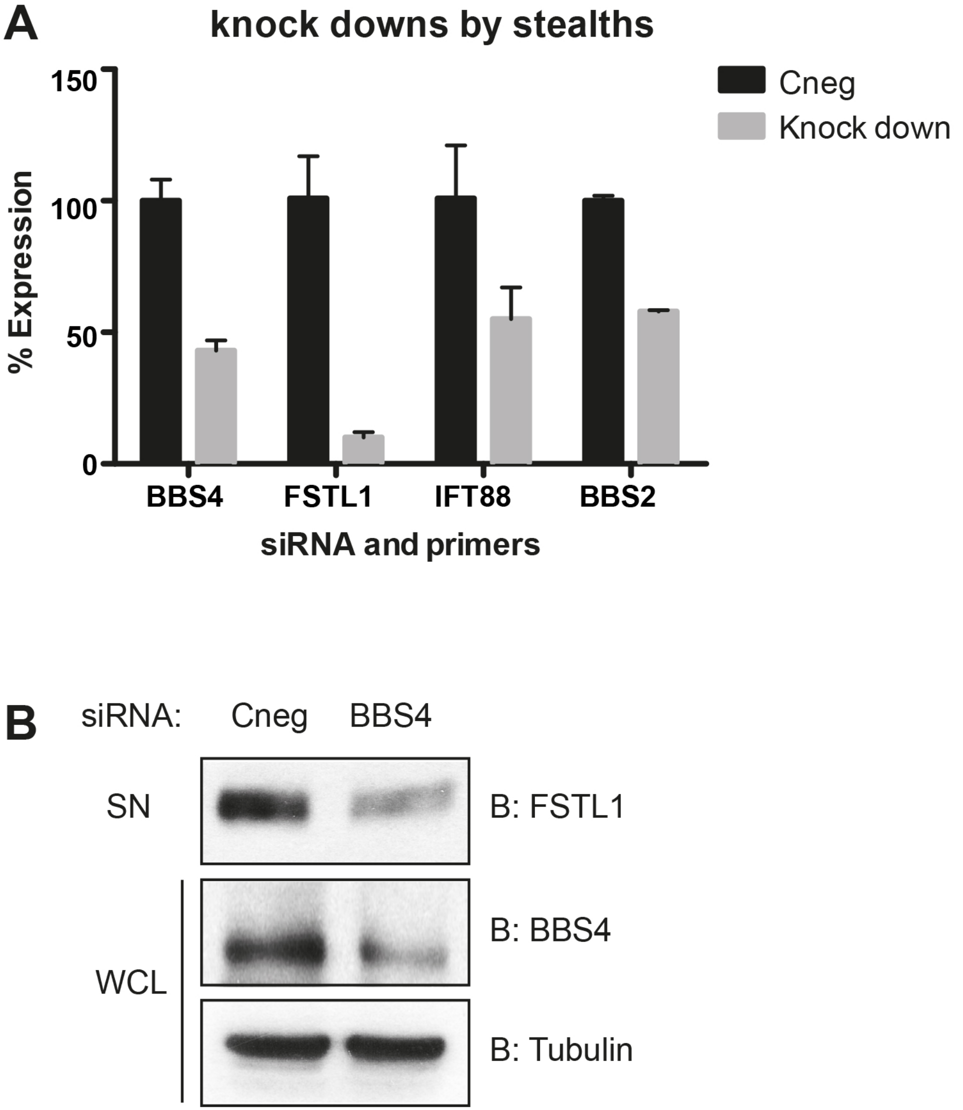

Figure S2: Validation of siRNAs used in this work.
(A) Quantitative RT-PCR was performed in hTERT-RPE1 cells transfected with the following siRNAs: Cneg, BBS4, FSTL1, IFT88 and BBS2 to analyze expression of the targeted gene. Gene expression is represented as % expression relative to control siRNA-transfected cells. **(B)** SN and WCL of BBS4 silenced cells were analyzed by western blot anti-BBS4 to evaluate knock down at the protein level.

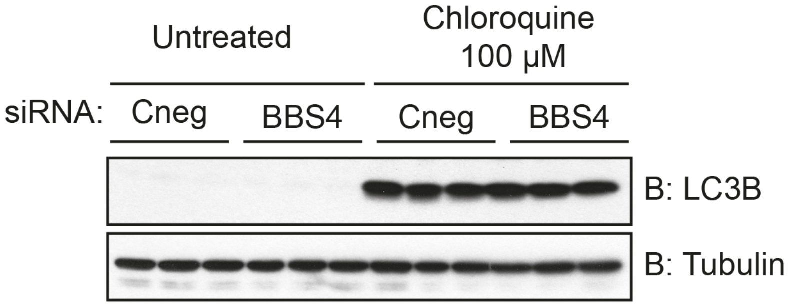

Figure S3: Chloroquine treatment inhibited lysosomes.
hTERT-RPE1 cells were transfected with siRNA Cneg, siRNA BBS4 (top panel) or siRNA IFT88 (bottom panel) during 24 hs and incubated with 100 μM chloroquine for an additional 24 hs. WCL were analyzed by Western blot anti LC3B to confirm the effective dose of chloroquine.

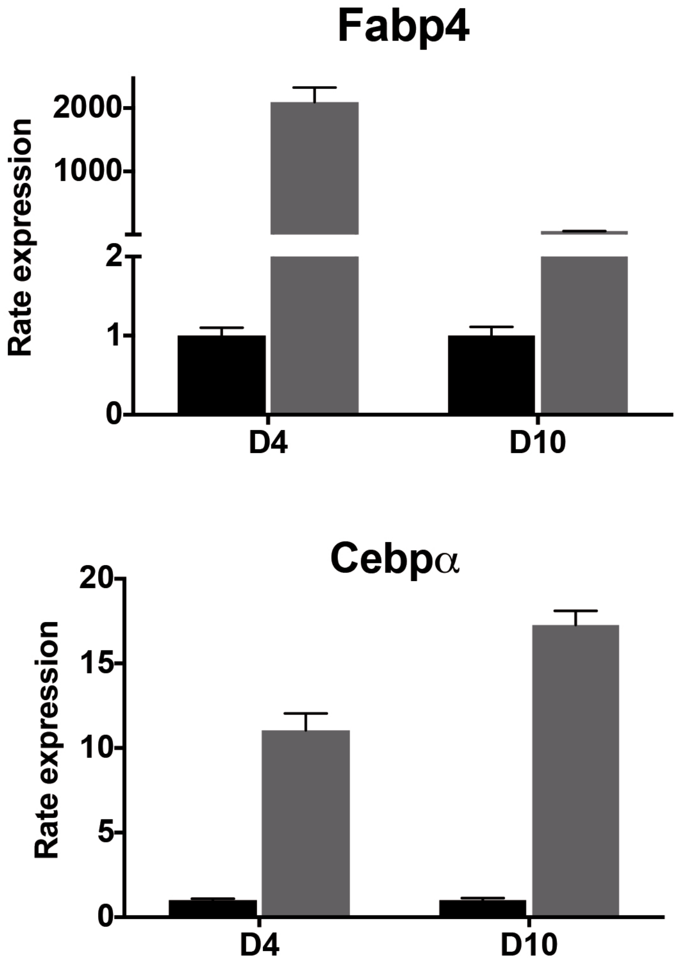

Figure S4: Adipogenesis markers gene expression is induced by differentiation treatment.
qRT-PCR analysis of gene expression of differentiation markers, Fabp4 and Cebpα in control 3T3-L1 cells at day 4 and day 10 of differentiation respectively. Bars represent the rate of expression of each gene relative to Gapdh. Gene expression in induced cells (grey bars) was normalized with uninduced cells (black bars) to compare fold change upon induction. Error bars represent standard deviation. The results shown are representative of multiple independent experiments (see also figures 6 and 7).

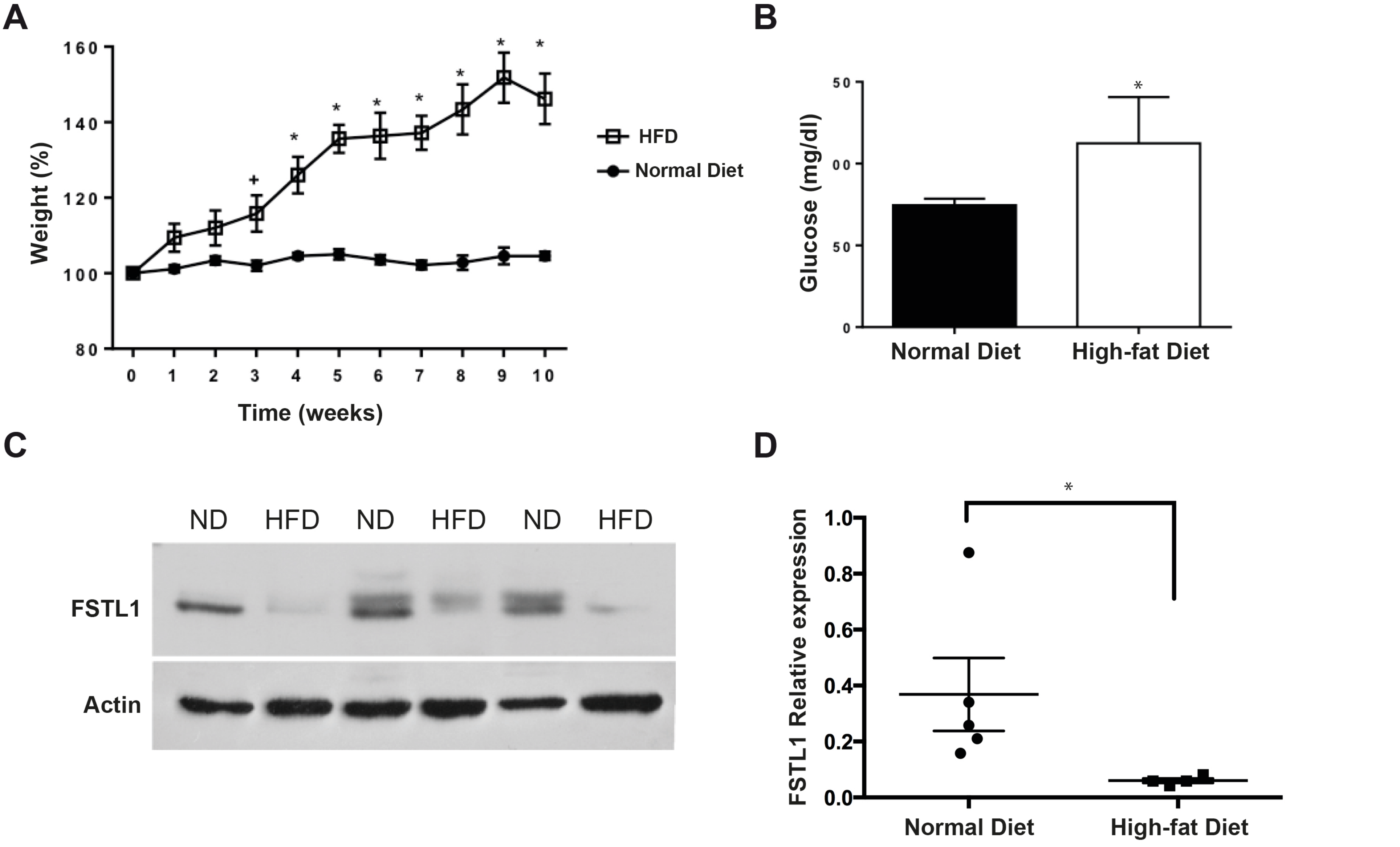

Figure S5: Fstl1 is downregulated in fat tissue of obese mice

(A) Weight gain in mice that were fed with standard chow (normal diet) or high-fat diet (HFD) for 10 weeks. **(B)** Glucose levels in blood measured at the end of the treatment. **(C)** Representative western blot of abdominal fat tissue of mice in normal diet (ND) or high-fat diet (HFD) for 10 weeks. **(D)** Densitometric analysis and quantitation of FSTL1 expression in abdominal fat (n=5 per group). * = P < 0,05, t-test.

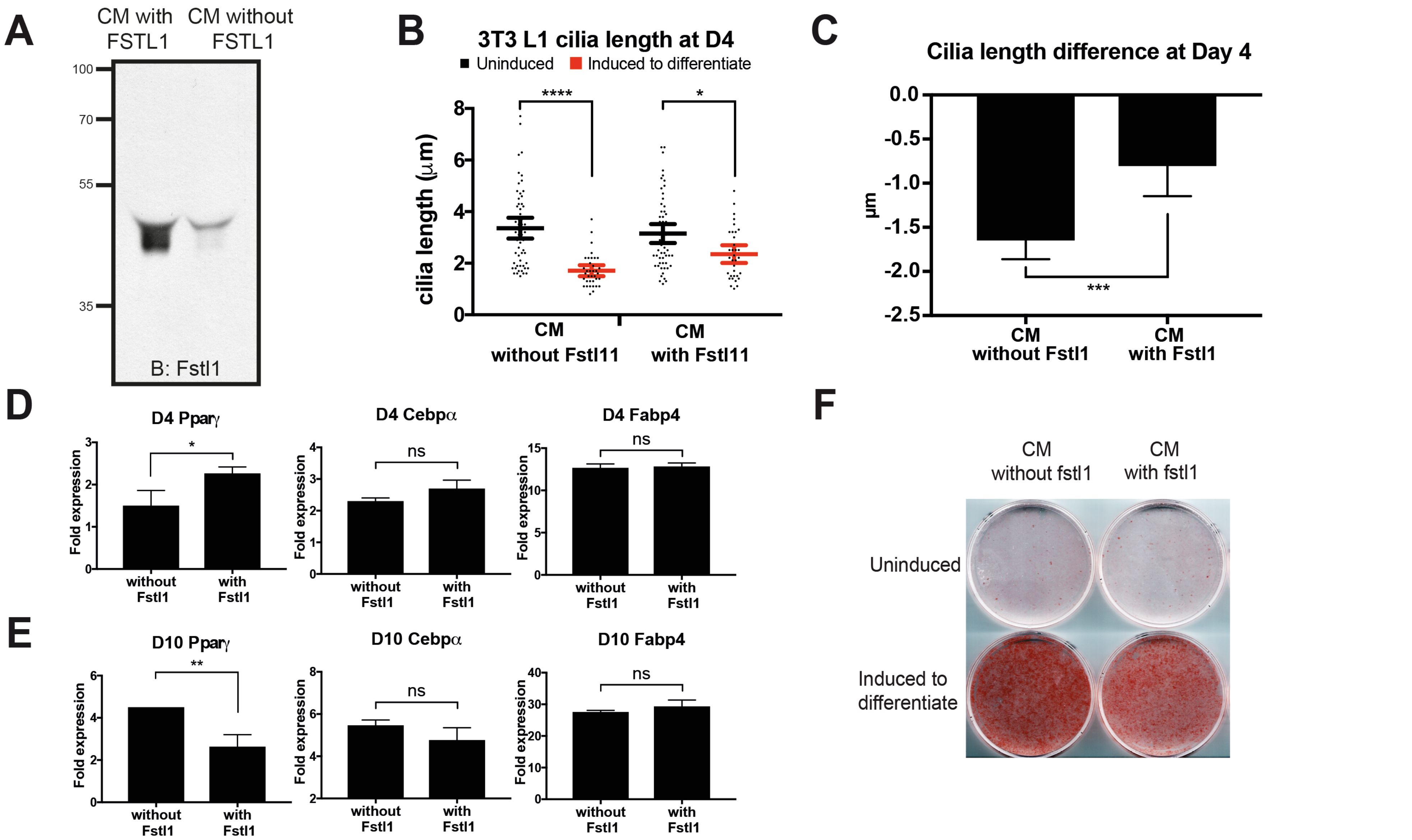

Figure S6: Maintaining Fstl1 levels high with conditioned media has a mild effect on 3T3-L1 preadipocyte differentiation.
(A) Conditioned media was collected from 3T3-L1 cells transfected with siRNA Cneg or siRNA Fstl1 and analyzed for Fstl1 content by Western blot anti-Fstl1. **(B)** Cilia length was measured in 3T3-L1 cells at day 4 of differentiation. Data are shown as a line at the mean and errors bars represent 95% confidence intervals. **(C)** Bar plot showing that the mean cilia length variation observed in (A) is milder in the presence of conditioned media containing Fstl1. Error bars represent standard deviation. **(D-E)** qRT-PCR analysis of gene expression of differentiation markers Pparγ, Cebpα and Fabp4 in 3T3-L1 cells at day 4 (D) and day 10 (E) of differentiation. Bars represent the fold change upon induction of each gene relative to Gapdh comparing induced cells with uninduced cells. Results shown are representative of two independent experiments. Error bars represent standard deviation. **(F)** Oil Red O staining of 3T3-L1 cells after 10 days of differentiation. The data shown are representative of two independent experiments. ns: P > 0.05; *: P = 0,01-0,05; **: P = 0,001-0,01; ***: P = 0,0001-0,001 and ****: P < 0,0001, ANOVA, or t-test.

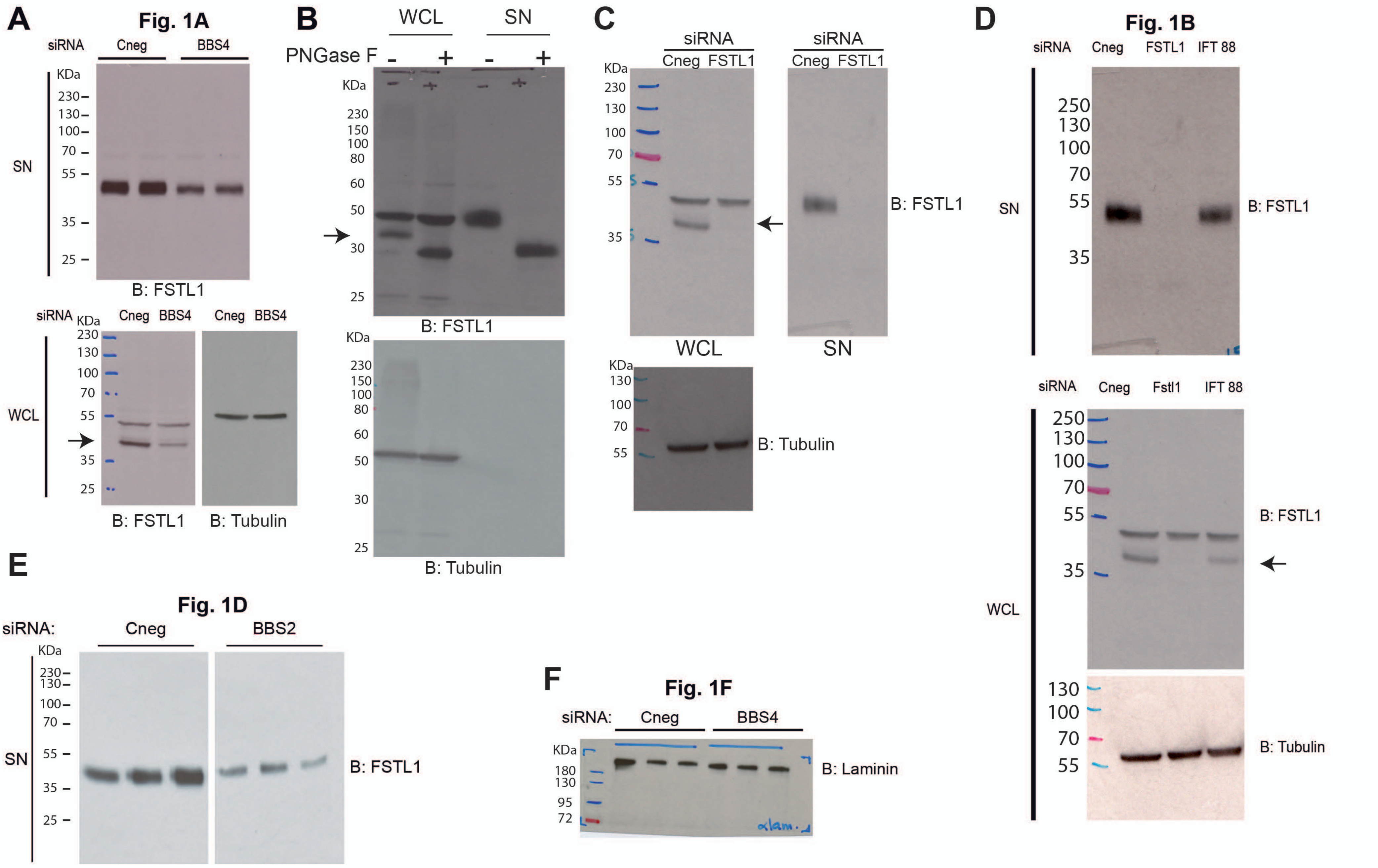

Figure S7: Full-length blots corresponding to Figure 1 and validation of FSTL1 antibody.
(A) Full-length blots corresponding to Figure 1A. Note that on cell lysates (WCL) the anti-human FSTL1 antibody recognizes mainly two bands. Our results show that the lower band corresponds to FSTL1 **(B and C). (B)** Treatment with the N-glycosydase PNGase F results in a shift of the lower band in WCL. **(C)** Knockdown of FSTL1 specifically results in the depletion of the lower band in WCL. **(D)** Full-length blots corresponding to Figure 1B. **(E)** Full-length blots corresponding to Figure 1D. **(F)** Full-length blots corresponding to Figure 1F. A representative full-length gel of Ponceau staining is shown in Figure S9.

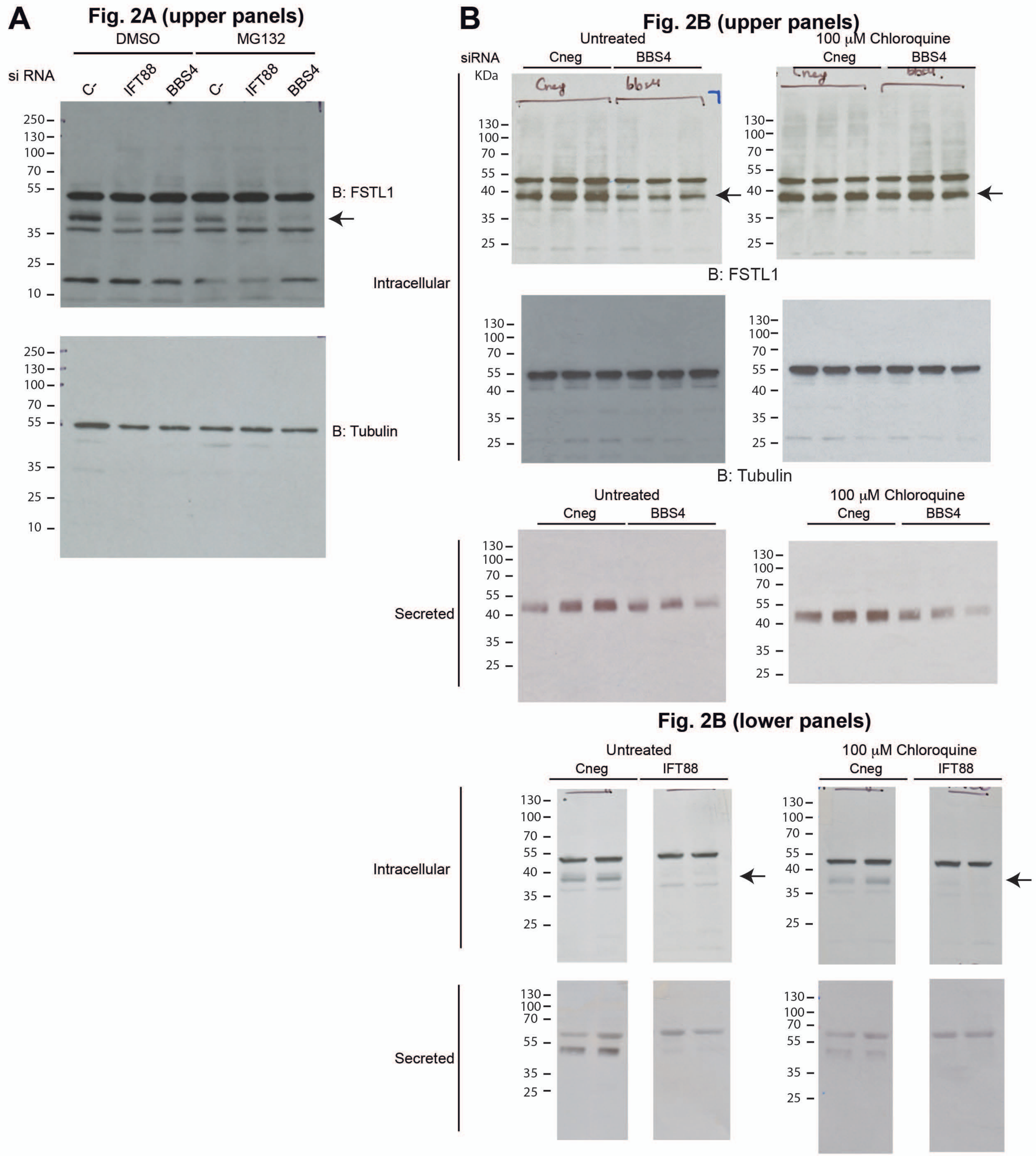

Figure S8: Full-lengh blots corresponding to Figure 2

(A) Full-length blots corresponding to Figure 2A upper panels. **(B)** Full length blots corresponding to Figure 2B: upper panels correspond to the BBS4 KD and lower panels to the IFT88 KD. A non-specific band of high molecular weigth is seen in the Western blot for secreted Fstl1 in IFT88 KD cells due to re-usage of the antibody.

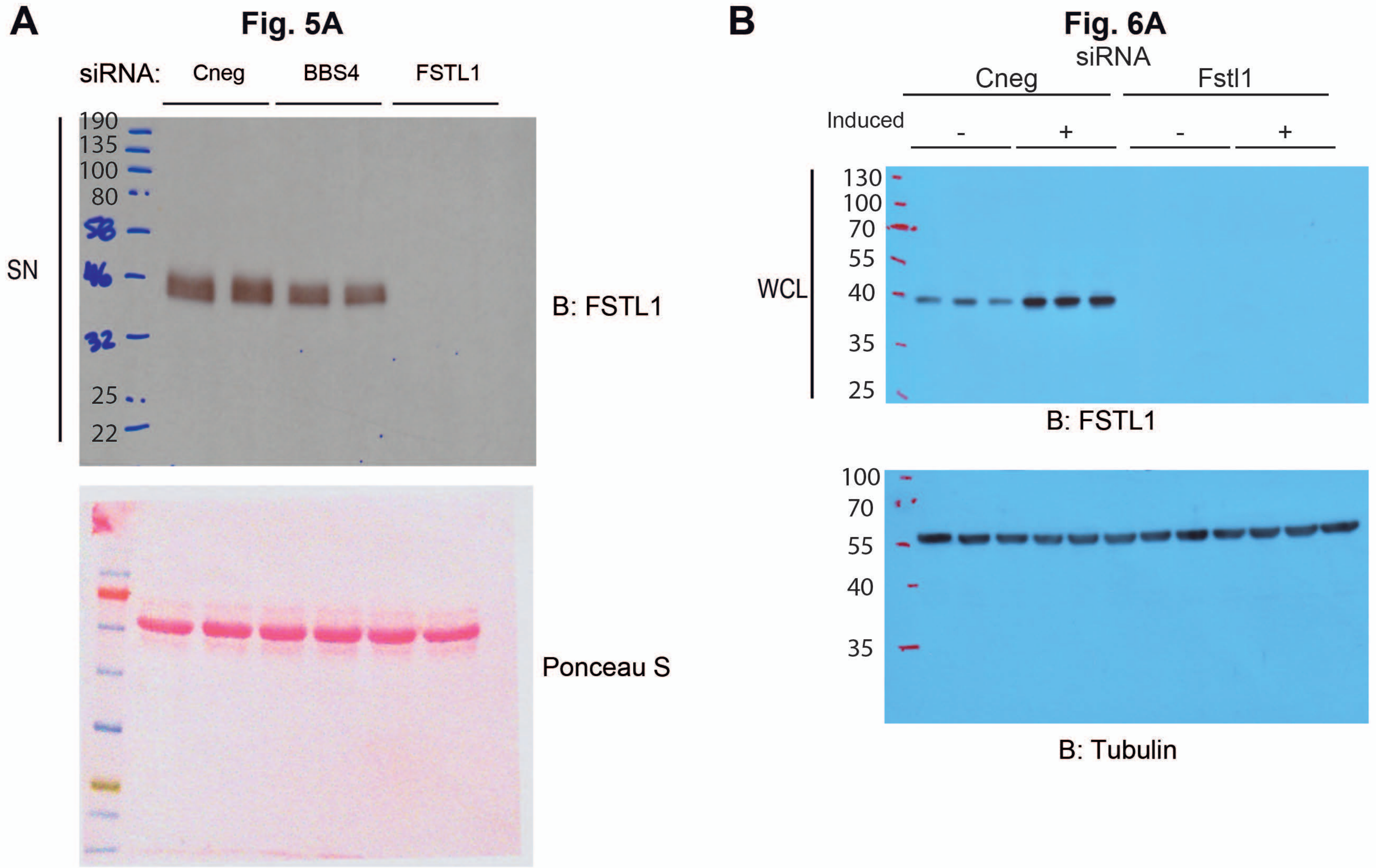

Figure S9: Full-length blots corresponding to Figures 5A and 6A.
(A) Full-length blot and gel corresponding to Figure 5A. A representative Ponceau staining is shown. **(B)** Full-length blots corresponding to Figure 6A. Note that the anti-mouse Fstl1 antibody used for Western blots in 3T3-L1 cells detects only one band that corresponds to Fstl1 (see knockdown).

Supplementary Table 1. Summary of percentage of ciliated cells and cilia length data presented in Figure 6.

		% Ciliated cells ± 95% CI		Cilia length (µm) ± 95% CI	
		siRNA Cneg	siRNA Fstl1	siRNA Cneg	siRNA Fstl1
Day 4	Uninduced	82 ± 7	50 ± 12	3.1 ± 0.2	2.2 ± 0.1
	Induced to differentiate	55 ± 7	46 ± 10	1.8 ± 0.1	1.9 ± 0.2
Day 10	Uninduced	75 ± 9	37 ± 9	2.4 ± 0.2	1.8 ± 0.2
	Induced to differentiate	15 ± 4	30 ± 7	1.7 ± 0.2	1.5 ± 0.1

Supplementary Table 2. Summary of percentage of ciliated cells and cilia length data presented in Supplementary Figure 6.

		% Ciliated cells		Cilia length (µm)	
		CM w/o Fstl1	CM w/Fstl1	CM w/o Fstl1	CM w/Fstl1
Day 4	Uninduced	71 ± 10	83 ± 9	3.4 ± 0.4	3.1 ± 0.4
	Induced to differentiate	42 ± 10	56 ± 11	1.7 ± 0.2	2.3 ± 0.3
Day 10	Uninduced	63 ± 14	57 ± 12	3.0 ± 0.3	3.0 ± 0.3
	Induced to differentiate	21 ± 8	38 ± 8	2.3 ± 0.4	2.9 ± 0.3

Supplementary Table 3. Summary of percentage of ciliated cells and cilia length data presented in Figure 7.

		Cilia length (µm) ± 95% CI	
		CM w/o Fstl1	CM w/Fstl1
Day 4	Uninduced	2.7 ± 0.1	2.7 ± 0.2
	Induced to differentiate	1.9 ± 0.1	2.7 ± 0.1
